# Aquatic Insects Transfer Pharmaceuticals and Endocrine Disruptors from Aquatic to Terrestrial Ecosystems

**DOI:** 10.1021/acs.est.0c07609

**Published:** 2021-03-02

**Authors:** Ana Previšić, Marina Vilenica, Natalija Vučković, Mira Petrović, Marko Rožman

**Affiliations:** † Department of Biology, Zoology, Faculty of Science, University of Zagreb, Rooseveltov Trg 6, 10000 Zagreb, Croatia; ‡ Faculty of Teacher Education, University of Zagreb, Trg Matice hrvatske 12, 44250 Petrinja, Croatia; § Catalan Institute for Water Research, Carrer Emili Grahit 101, 17003 Girona, Spain; ∥ Catalan Institution for Research and Advanced Studies (ICREA), Barcelona, Spain; ⊥ Ruđer Bošković Institute, Bijenička cesta 54, 10000 Zagreb, Croatia

## Abstract

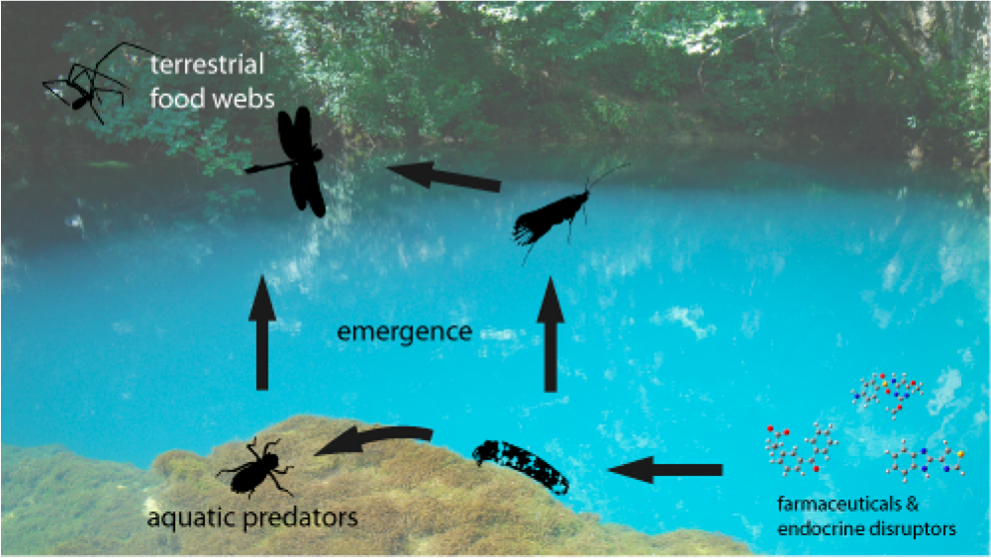

A wide range of pharmaceuticals and endocrine disrupting compounds enter freshwaters globally. As these contaminants are transported through aquatic food webs, understanding their impacts on both aquatic and terrestrial ecosystems remains a major challenge. Here, we provide the first direct evidence of the transfer of pharmaceuticals and endocrine disruptors through the aquatic–terrestrial habitat linkage by emerging aquatic insects. We also show that the type of insect metamorphosis and feeding behavior determine the bioaccumulation patterns of these contaminants. Adult Trichoptera, an important food source for riparian predators, showed an increased body burden of pharmaceuticals and endocrine disruptors. This implies that terrestrial predators, such as spiders, birds, and bats, are exposed to mixtures of pharmaceuticals and endocrine disruptors of aquatic origin, which may impact their physiology and population dynamics. Overall, our study provides valuable insights into the bioaccumulation patterns and trophic cross-ecosystem transfer of these contaminants, from aquatic primary producers to terrestrial predators.

## Introduction

A wide range of emerging contaminants (ECs) enter freshwater ecosystems through urban, industrial, and agricultural wastewater effluents. In spite of the variety of advanced treatment options available, the majority of wastewater treatment plants only remove a fraction of ECs such as pharmaceuticals (PhACs) and endocrine disrupting compounds (EDCs).^1−3^ The fate, behavior, and transport of ECs through aquatic ecosystems depend on the interplay of many physical, chemical, and biological processes.^4−7^ In spite of the growing number of studies addressing this topic, the evaluation of ecological impacts associated with the presence of ECs in aquatic ecosystems remains a major challenge.^8^ Gaining an understanding of the ecological impacts of chronic exposure to complex and variable mixtures of ECs, the only environmentally relevant scenario, is particularly challenging.^9^

Bioaccumulation of PhACs and EDCs (i.e., the process in which these substances are absorbed in an organism by all routes of exposure, dietary, and ambient environment^10^), has been observed at both lower and higher trophic levels in aquatic food webs, e.g., from biofilm to predatory invertebrates^11^ and fish.^12^ Both bioaccumulation and bioconcentration of PhACs (i.e., the process when ECs are absorbed from the ambient environment only through respiratory and dermal surfaces)^10^ were shown to be species and compound specific.^13,14^ First insights into the trophic transport of these ECs in aquatic food webs indicate that lower level consumers are the main receivers of PhACs;^14^ however, knowledge on potential trophic biomagnification is very limited. Generally, the biomagnification of contaminants is especially problematic, considering observed adverse effects in higher consumers.^15^ Food webs are usually interconnected across habitat boundaries, and aquatic and terrestrial ecosystems are linked through emerging aquatic insects transporting resources and energy from freshwaters to adjacent terrestrial habitats.^16^ Some organic contaminants, such as polychlorinated biphenyls (PCBs), experience bioamplification or an increase in body burden during the metamorphosis of emerging aquatic insects, hence resulting in an elevated exposure risk of terrestrial riparian predators, i.e., potential trophic biomagnification.^17^ For PhACs, hitherto only one field study has suggested trophic transfer through the aquatic–terrestrial habitat linkage (ATHL) by measuring similar concentrations of different compounds in aquatic insect larvae and riparian spiders.^18^ Although the effects of aquatic contaminants can be propagated across ecosystem boundaries by aquatic insects, the mechanisms driving these are poorly understood, despite the high ecological relevance for the terrestrial ecosystem.^17,19^

To provide the first direct evidence of transfer of PhACs and EDCs through the ATHL by emerging aquatic insects, we conducted a laboratory microcosm experiment, along with a field study. In the microcosm experiment, two Trichoptera species (caddisflies) were exposed to a mixture of four PhACs, the nonsteroidal anti-inflammatory drugs (NSAIDs) diclofenac and ibuprofen, the antihistamine diphenhydramine, and the lipid regulator gemfibrozil, and two EDCs, the preservative methylparaben and the organophosphate flame retardant tris(2-butoxyethyl)phosphate (TBEP), over a 65-day period. The selected ECs were quantified in the samples of aquatic larvae and emergent adults, as well as in water and moss (the major food source for the insects), allowing us to assess the importance of trophic vs aqueous uptake of different ECs in aquatic insects. During the *in situ* collections, water, biofilm samples, and aquatic and terrestrial stages of two aquatic insect orders (Odonata and Trichoptera) and riparian spiders were collected from five sites affected with wastewater effluents and agricultural runoff. The samples were then screened for a total of 143 ECs (119 PhACs and 24 EDCs) using a targeted ultraperformance liquid chromatography system coupled to a tandem mass spectrometer (LC/MSMS) analysis. Integrated results provide valuable insights into compound- and taxa-specific bioaccumulation patterns, as well as the fate of PhACs and EDCs transfer through ecosystem boundaries via emerging aquatic insects.

## Materials and Methods

### Microcosm Experiment: Experimental Design and Sample Collection

We conducted the microcosm experiment with a simplified freshwater food web containing nonvascular macrophytes (or moss; Bryophyta) and the larvae of two Trichoptera taxa (*Drusus croaticus* Marinković-Gospodnetić and *Potamophylax* Wallengren, Limnephilidae) feeding as shredders and grazers. Trichoptera larvae, water, sand, and stones were collected from a pristine spring reach (Majerovo vrelo, Gacka River; N44.81474 E15.35856, on May 06, 07 2018) upstream of any anthropogenic impacts. Upon collection, 15 microcosms (aquaria of 30 × 20 × 15 cm) were installed with 3 L of water each, and equal amounts of sand (10 tablespoons), stones (3 stones >10 cm and 10 stones 2–5 cm), mixtures of moss species (total of 3 tufts of 6–8 cm in diameter and length of the plants up to 15 cm; *Cinclidotus aquaticus* (Hedw.) Bruch and Schimp., *Leptodictyum riparium* (Hedw.) Warnst., *Rhynchostegium riparioides* (Hedw.) Cardot), and larvae of both Trichoptera species (ca. 30 larvae per species). Four microcosms were assigned as controls, and 11 were exposed to a mixture of ECs, all randomly placed in 3 incubators (POL-EKO APARATURA, Poland). Constant oxygen levels were kept using aquaria air pumps, and to minimize evaporation, each microcosm was covered with a glass cover. The temperature was kept constant at 9.3 °C for the first 20 days and successively increased 0.1 °C every 15 days, mimicking the thermal regime of the spring Majerovo vrelo (S. Gottstein, unpublished data). All microcosms were acclimatized for 7 days, and subsequently, 11 of them were daily exposed to a mixture of 6 ECs over a 65-day period: 4 PhACs, diclofenac, ibuprofen, diphenhydramine, and gemfibrozil, and 2 EDCs, methylparaben and tris(2-butoxyethyl)phosphate (TBEP). The volume of water was kept constant by adding fresh dechlorinated tap water (ca. 200 mL every 2 weeks), and the concentration of each compound was kept at a pseudoconstant concentration of 500 ng L^–1^.

Water, moss, and Trichoptera larvae were sampled 4 times: after the acclimatization period—day 0, day 21, day 35, and day 65. At each sampling date, replicate samples were taken from each microcosm (25 mL of water, 2 g of moss, and 3–14 Trichoptera larvae of each species); however, these were pooled per treatment per species to minimize the variability between the microcosms, and analytical replicates for each sampling date were taken. Additionally, emerging adult Trichoptera were collected as they emerged (i.e., emerged, flying specimens were collected daily, days 40–65). Trichoptera larvae were kept in clean aquaria for 24 h to allow for gut clearance prior to collection,^20^ and then samples were freeze-dried and stored at −80 °C until further processing.

### *In Situ* Sample Collection

Water, biofilm samples, and aquatic and terrestrial stages of two aquatic insect orders (Trichoptera and Odonata) and riparian spiders were collected from five sites in NW Croatia that exhibited a gradient of pollution (wastewater effluents and agricultural runoff, Table S1). At each site, two collections within maximally 30 days were executed, in order to collect aquatic (larval) and terrestrial (adult) stages of insects inhabiting the targeted sites. Water and biofilm samples were taken in replicates; water was collected in 1 L bottles, and biofilm was scraped off from stones. Adult insects were collected with an entomological net, and spiders were collected from overhanging riparian vegetation by hand. Aquatic insect larvae were collected with a D-net, and all microhabitats were screened. Upon collection, aquatic insect larvae were kept in 10 L containers with river water and transported to the lab, where they were placed in river water from the respective collection site for 24 h to allow gut clearance. Taxa were separated on their respective species/genera/family (Table S1), freeze-dried, and stored at −80 °C until further processing.

### Analysis of PhACs and EDCs in Water and Biota Samples

Water samples were processed using methods described in detail in Gros et al.^21^ Biota samples were processed using modified methods of Previšić et al.^22^ Briefly, 1.5 mL of ice cold acetonitrile was added to 50 mg of freeze-dried biota tissue. At this point, a standard mixture containing all isotopically labeled standards was added as an internal standard. Tissue was lysed by bead beating in a home-built bead beater with 2.3 mm diameter chrome-steel beads at a frequency of 20 Hz for 5 min at 4 °C. Samples were centrifuged at 20 000*g* for 10 min, and supernatant 1 was collected. Remaining pellet was resuspended in 1.5 mL of ice cold acetonitrile, and additional lysis was done via ultrasonic probe (Sonoplus HD4050, Bandelin Electronic GmbH, Germany) for 1 min at 50% of intensity. Samples were vortexed for 5 min and centrifuged at 20 000*g* for 10 min, and supernatant 2 was collected. Supernatants 1 and 2 were evaporated to dryness and dissolved in 1 mL of water with EDTA at 1%.

Both water and biota samples were additionally cleaned with solid phase extraction using Waters Oasis HLB cartridges (60 mg, 3 mL). Cartridges were conditioned with 3 mL of acetonitrile followed by 3 mL of HPLC-grade water at a flow rate of 1 mL min^–1^. One hundred milliliters of water sample or 2 mL of biota sample extracts was loaded at 1 mL min-1. Sample were washed with 1 mL of water and consequently extracted with 1.5 mL of pure acetonitrile at a flow rate of 1 mL min^–1^. Final extracts were evaporated to dryness under a gentle nitrogen stream and reconstituted with methanol/water (50:50, v/v) and used for targeted analysis.

Target analysis was performed using an ultraperformance liquid chromatography (UPLC) system (Waters Milford, USA) coupled to a hybrid quadrupole linear ion trap mass spectrometer Qtrap 5500 (Applied Biosystems, USA). Details regarding UPLC separation can be found in Supporting Information S1, while instrument-dependent and scheduled MRM parameters are summarized in refs (21) and (23). The sample volume injected was 5 mL for all analyses. Samples of the microcosm experiment were screened for 2 EDCs and 4 PhACs, whereas samples of *in situ* collections were screened for 119 PhACs and 24 EDCs. A list of all compounds is provided in Table S2 of the Supporting Information. Instrument control, data acquisition, and data analysis were carried out using Analyst 1.5.1 software (Applied Biosystem). Target compounds were quantified using an internal standard method by the Bquant script for batch quantification of liquid chromatography mass spectrometry data using the procedure described in ref (24).

### Statistical Analysis

#### Bioaccumulation of ECs in the Microcosm Experiment

The bioaccumulation patterns of individual PhACs and EDCs in moss and Trichoptera were tested using the nonparametric repeated measures tests (Wald and ANOVA tests) implemented in the nparLD package in R.^25^ The package was used to test overall differences in patterns with respect to treatment, time of exposure, and species and their interactions, as well as for conducting pairwise comparisons between time points for each individual Trichoptera species and moss.

In order to assess influence of physicochemical properties on the uptake of ECs, the bioaccumulation factor (BAF) was calculated. The BAF (in L/g) was calculated as the ratio of EC concentration in the organism (ng/g) to freely dissolved chemical concentration in the water (ng/L). It is important to note that this study was not designed to assess the BAF but to demonstrate trophic cross-ecosystem transfer of ECs. Accordingly, a tight steady state criterion, i.e., no significant differences between three sequential sampling periods during the uptake phase with a consistent aqueous exposure concentration, was not achieved.^10^ Instead, pseudo-steady state was assumed at the maximal observed PhACs concentration in the organism. Physicochemical properties of individual ECs used to evaluate correlation with the BAF were compiled using the National Institutes of Health (Maryland, USA) PubChem open chemistry database and DrugBank Online (University of Alberta, CA). The most widely used descriptors—the octanol–water partition coefficient (log *K*_ow_), polar surface area (PSA), relative molecular mass (Mr), and aqueous solubility (log *S*)—were used (Table S2). In addition, a octanol–water distribution coefficient (log *D*) that considers the ratio of all EC molecular entities was considered (Table S2). The fraction of molecular entities at the pH of 8.5 (mean value in the experiment) was estimated using ECs p*K*_a_ value and the log *K*_ow_ of ionic species by using following relationship log *K*_ow_(ion) = log *K*_ow_(neutral) – 3.5.^26^

#### Bioaccumulation of ECs *In Situ*

Relationships between compounds (PhACs and EDCs) and biota were visualized using the graph theory. A network was created in a way that compounds and biota were used as nodes with edges connecting compounds to the biota in which they are found. In order to get a better understanding of the possible organization of nodes in clusters, detection of community structure was attempted using the modularity maximization method. Graphic representation as well as community detection was done with the Wolfram Mathematica technical computing program (version 10, Wolfram Research, U.K.).

Differences in concentrations of EC totals (total ECs, total PhACs, and total EDCs) and of individual compounds were tested for data encompassing all five sampling sites at three different levels (data pooled across all sites). For overall differences in concentrations between taxa and habitats (aquatic/terrestrial), a Mann–Whitney U test was used between the following: (I) aquatic insects (Odonata and Trichoptera; aquatic and terrestrial stages) and riparian spiders, (II) aquatic larvae and terrestrial adults of Odonata, and (III) aquatic larvae and terrestrial adults of Trichoptera. All Mann–Whitney U tests were conducted in Statistica 10.0 (StatSoft, Inc.).

In order to infer patterns of ECs trophic transfer within each habitat and through the ATHL, a data set from three sites (BI, PR, and VZ, Table S7) with a simplified food web was compiled, including three aquatic compartments: water, biofilm, Trichoptera larvae (grazing or grazing and shredding;^27^ as aquatic prey^28^), and two terrestrial: adult Trichoptera (as terrestrial prey^29^) and terrestrial predators (riparian spiders^30^ at PR and VZ and adult dragonflies [Odonata, Anisoptera^16^] at BI). Data for each compartment were pooled across all three sites. Differences in concentrations of EC totals (total ECs, total PhACs, and total EDCs) and of individual compounds between five compartments were tested using the Kruskal–Wallis ANOVA by ranks and median tests and multiple comparisons tests applied in Statistica 10.0 (StatSoft, Inc.). For the same data set, bioamplification factors (BAmF) and biomagnification factors (BMF) were also calculated. BAmFs were calculated as the ratio of the mean concentration of ECs in adult Trichoptera to the mean concentration of ECs in Trichoptera larvae collected at a given site.^31^ BMFs were calculated as the ratio of the mean concentration of ECs in each higher trophic category to the mean concentration of ECs in each lower trophic category for both habitats, i.e., Trichoptera larvae and biofilm for the aquatic habitats and terrestrial predators and Trichoptera adults for the terrestrial habitats. In cases where particular ECs were measured only in the higher trophic category and/or adult Trichoptera at all three sites, a factor of >1 was assigned (denoted in figure captions).

## Results

### Bioaccumulation and Transfer of ECs through the ATHL via Emerging Insects in the Microcosm Experiment

**Figure 1:**
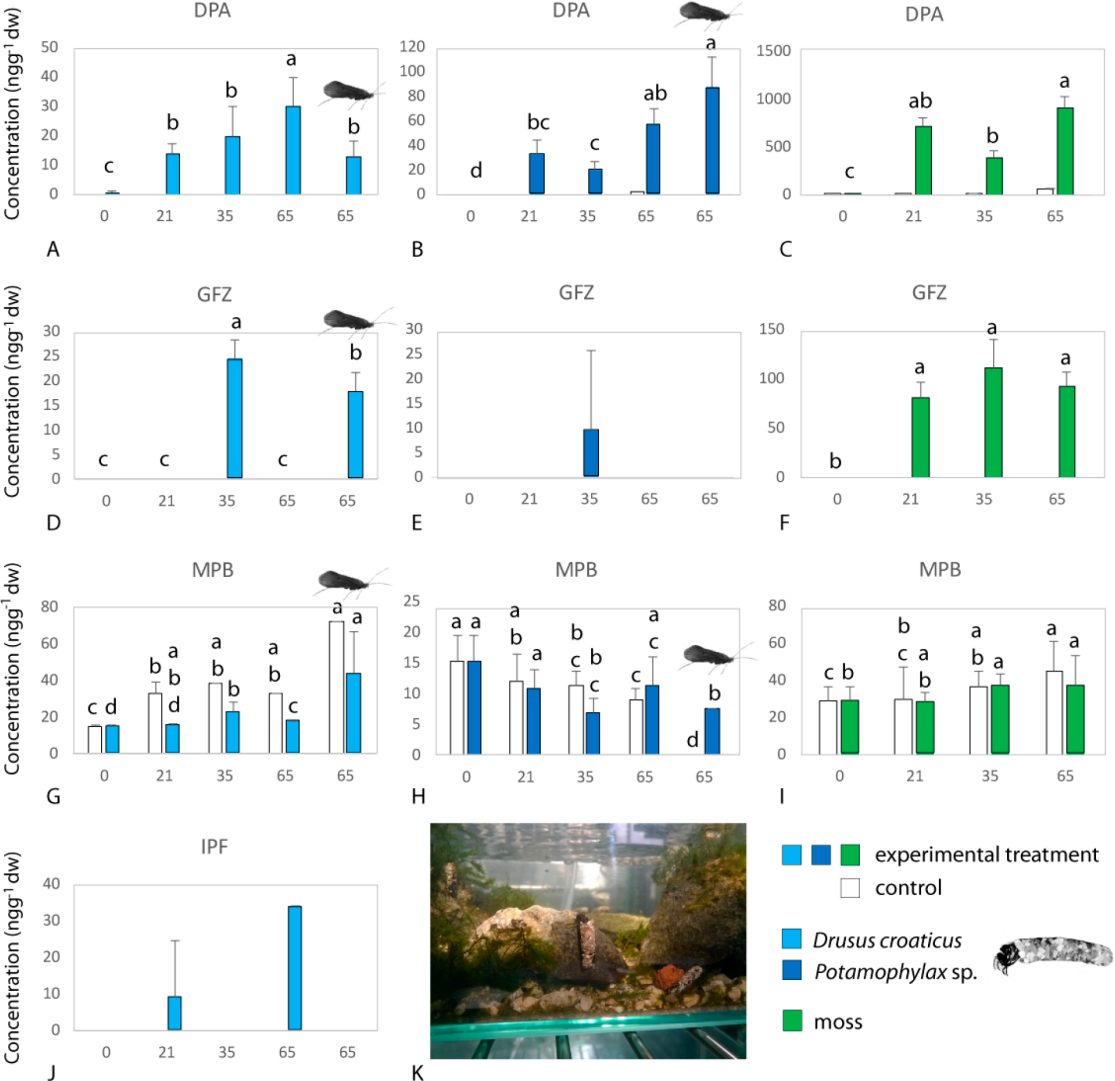
Concentration of pharmaceuticals (PhACs) and endocrine disrupting compounds (EDCs) in moss and Trichoptera in the microcosm experiment. Transport from aquatic to terrestrial ecosystems is observed as PhACs and EDCs are measured in emerging adult insects (noted with the figure of the adult Trichoptera). Concentration is shown in ng g^–1^ of dry tissue weight (dw) on the *y*-axis, whereas the *x*-axis shows the number of days of treatment; 0–65 larvae, 65 also adult Trichoptera. Two Trichoptera taxa were included in the microcosm experiment, *Drusus croaticus* and *Potamophylax* sp. PhACs: DPA—diphenhydramine (A–C); GFZ—gemfibrozil (D–F); IBP— ibuprofen (J). EDC: MPB—methylparaben (G–I). The significance of nonparametric Wald and ANOVA tests and multiple comparisons tests is listed in Tables S3 and S4.

Results of the microcosm experiment provide the first direct evidence of PhACs transport from aquatic to terrestrial ecosystems through emerging insects. Out of six compounds in the experiment, bioaccumulation was measured for four compounds in Trichoptera larvae (PhACs: diphenhydramine, gemfibrozil, and ibuprofen; EDC: methylparaben) and for two compounds in adults (diphenhydramine, gemfibrozil; Figure 1), as revealed through significant differences between the control and experimental treatments (nonparametric Wald and ANOVA tests; Table S3). In addition, in diphenhydramine and gemfibrozil significant differences were observed in the duration of exposure, as well as the interaction treatment*duration of exposure (Wald and ANOVA tests; for diphenhydramine, treatment: WT and AT *p* < 0.0001; duration of exposure: WT *p* < 0.0001; AT *p* < 0.001; gemfibrozil, treatment: WT and AT *p* < 0.001, duration of exposure: WT *p* < 0.0001; Table S3). No significant bioamplification, i.e., increase in concentration during insect emergence, was observed for any of the compounds (multiple comparisons tests, Table S3).

### Aqueous and Dietary Accumulation Contribute to Bioaccumulation of ECs in Insects

All detected compounds resulted in measurable concentrations in larval Trichoptera tissues after 21 days of exposure to a pseudoconstant concentration of 500 ng/L, with the exception of gemfibrozil (measured after 35 days, Figure 1). Similarly, the accumulation of all compounds in the experiment except ibuprofen was recorded in moss tissues after 21 days of exposure (Figure 1). Therefore, the presence of ibuprofen in Trichoptera larvae implies aqueous exposure as the main uptake route in aquatic invertebrates, i.e., bioconcentration. Diphenhydramine was the compound with the highest concentration in moss tissues and the only compound showing a strong positive relationship of the bioaccumulation pattern in moss and both Trichoptera species in the experimental treatment (Spearman’s rank correlation; *r* = 0.859, *p* = 0.0001 and *r* = 0.762, *p* = 0.004, for *Potamophylax* sp. and *D. croaticus*, respectively), indicating that the main uptake route of this compound is through diet. This finding is to our knowledge the first direct evidence of dietary transfer of PhACs from moss to aquatic insect larvae. Even though bioaccumulation of gemfibrozil was observed in both Trichoptera larvae and moss tissues (Wald and ANOVA tests; treatment, WT and AT *p* < 0.0001; time, WT *p* < 0.0001, AT *p* < 0.0001, Figure 1; Tables S3 and S4), no correlation in bioaccumulation patterns was observed. Hence, the main source of exposure is most likely aqueous or a combination of dietary and aqueous accumulation. Methylparaben was measured in both Trichoptera taxa and moss over the whole course of the experiment in both the control and experimental treatments (Figure 1G, H, and I); however, there were differences between treatments only in the case of Trichoptera larvae (Wald and ANOVA tests for Trichoptera; treatment WT and AT *p* < 0.05, Table S3). Thus, even though pristine spring biota was collected for the experiment, methylparaben seemed to be already accumulated in tissues. Anthropogenic contamination of the selected spring biota was highly unlikely; hence, our results indicate a natural origin of this compound in the current study. Moreover, they highlight the need for more detailed knowledge on the physiology of both aquatic plants^32^ and invertebrates,^33^ in order to realistically assess the bioaccumulation of parabens *in situ*,^22^ as well as the potential role and impacts these compounds have on biota.^34^

### Bioaccumulation of ECs in Trichoptera Is Species- and Compound-Specific

Bioaccumulation showed differing trends with respect to both specific compounds and Trichoptera species. Ibuprofen only accumulated in *D. croaticus* larvae (Figure 1J), while gemfibrozil was detected in the larvae of both species but only in *D. croaticus* adults (Figure 1D and E). Diphenhydramine had the highest mean concentrations in *D. croaticus* larvae and in *Potamophylax* sp. adults, whereas in methylparaben it was the opposite (*Potamophylax* sp. larvae and *D. croaticus* adults; however, this has to be treated with caution–see above, Figure 1A, B, G, and H). Hence, for all three PhACs, significant differences between the two species were inferred by the Wald and ANOVA tests (diphenhydramine: WT and AT *p* = 0.0338, gemfibrozil, ibuprofen: WT and AT *p* < 0.0001; Table S3). Differences in the bioaccumulation of diphenhydramine between two Trichoptera taxa belonging to the same family are most likely related to differences in their feeding behavior; i.e., *D. croaticus* is predominantly a grazer, whereas *Potamophylax* larvae are mainly shredders and partially grazers.^35^

Correlation between physicochemical descriptors and BAF values was done only for *D. croaticus* larvae since the bioaccumulation of minimum three ECs was observed. The results do not indicate statistically significant correlation between physicochemical descriptors and BAF values (Figure S1). Spearman’s rank correlation coefficient showed vague positive correlation with log *S* and negative correlation with log *K*_ow_, log *D*, PSA, and *M*_r_ (Figure S1).

### Aquatic Insects Transport ECs through Food Webs Linking Aquatic and Terrestrial Ecosystems—Insights from *In Situ* Collections

**Figure 2:**
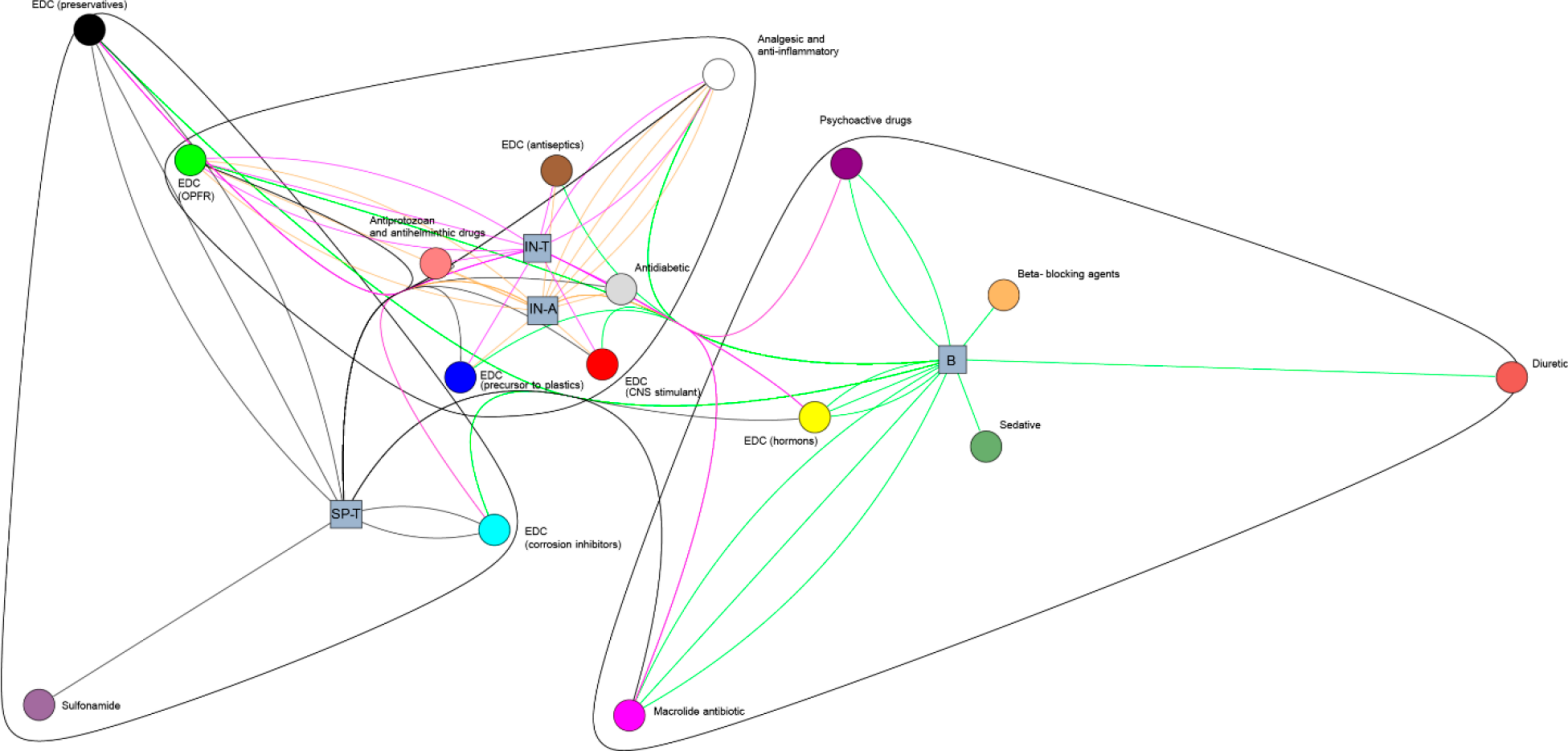
Pairwise relationships between compounds (pharmaceuticals and endocrine disrupting compounds—EDCs) and biota and their connections as detected by the graph community. Compounds are represented by circles and biota as squares (B— biofilm, IN-A—insects aquatic stages, IN-T—insects terrerstrial stages, SP-T—spiders). Using the information encoded in the graph topology, three clusters related to (I) biofilm, (II) aquatic and terrestrial insect stages, and (III) riparian spiders were identified.

Samples from 5 sites were screened for 126 PhACs and 25 EDCs. A total of 40 different compounds were measured in water, 26 compounds in the biofilm, 20 in terrestrial insect stages and riparian spiders, and 19 in aquatic insect larvae (Table S5). All compounds quantified in both aquatic and terrestrial insect samples were also detected in water and biofilm, suggesting aquatic and/or dietary accumulation routes. Pairwise relationships between compounds (PhACs and EDCs) and biota were analyzed using the graph theory. Both compounds and biota were used as nodes with edges connecting compounds to the biota in which they were found, thus visualizing their connections. By only using the information encoded in the graph topology, the graph community detection identified three clusters related to (I) biofilm, (II) aquatic and terrestrial insect stages, and (III) riparian spiders (Figure 2). Such topology illustrates the hierarchical nature of the transport path of ECs through the ATHL, related to both trophic relationships and aquatic insect emergence. The dense internal connection between aquatic and terrestrial insect stages further suggests the direct transport of ECs from aquatic to terrestrial ecosystems through emerging insects. Together with compounds that are transported through the food web, the structure of the biofilm cluster revealed hydrochlorothiazide (diuretic), azaperol (sedative), and metoprolol (beta-blocking agent) as the compounds accumulated in biofilm but not transported into higher trophic levels. Similarly, the terrestrial spiders cluster revealed the antibiotic sulfadimethoxine as the compound only detected in riparian spiders, implying a potentially different source of exposure or environmental fate.

### Ecosystem Transfer of Contaminants Is Life History Dependent

**Figure 3:**
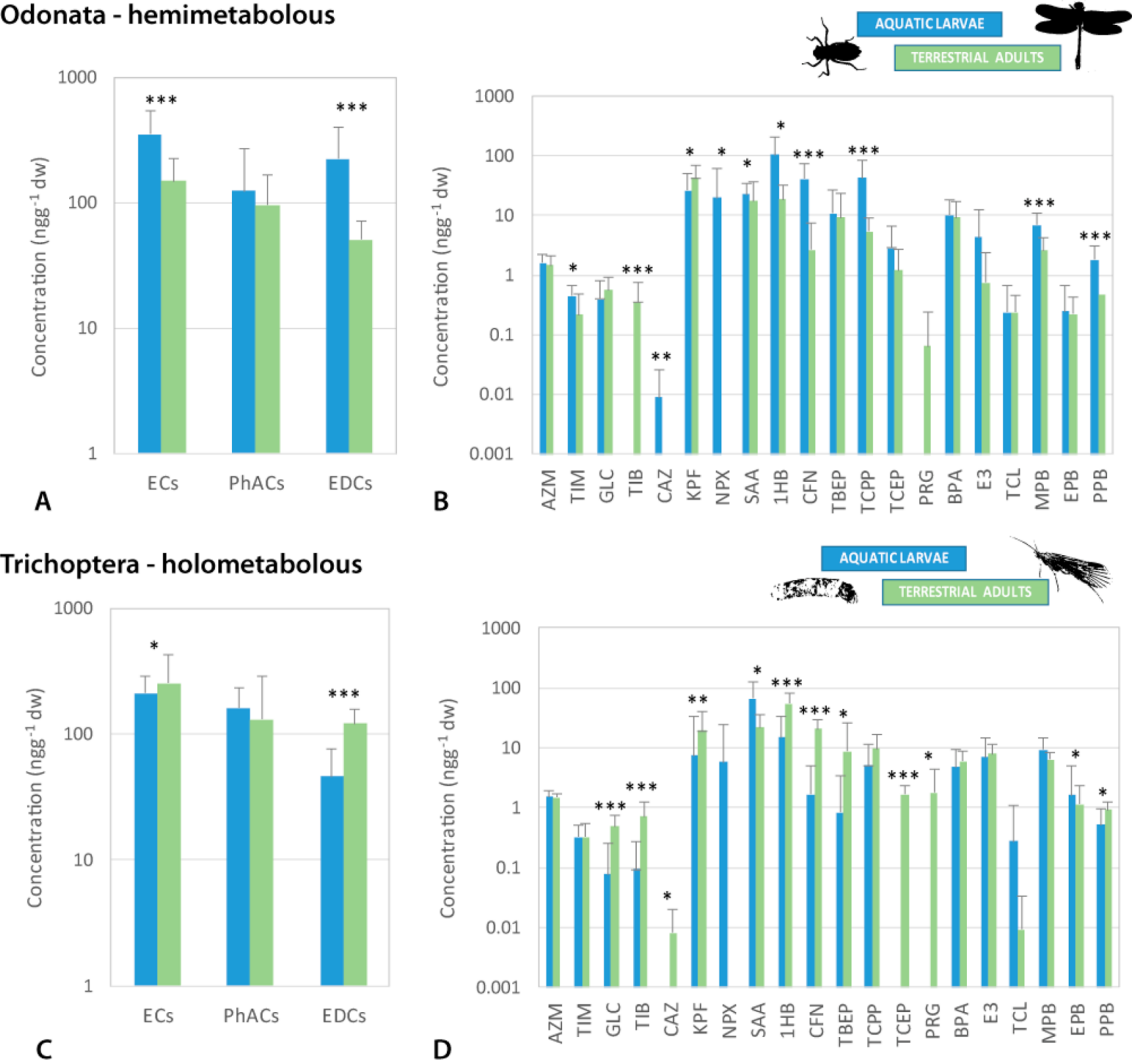
Total concentrations of emerging contaminants (ECs), pharmaceuticals (PhACs), and endocrine disrupting compounds (EDCs) (A and C) and concentrations of individual compounds (B and D) in aquatic and terrestrial stages in Odonata and Trichoptera measured *in situ* at five sites in NW Croatia. Concentrations are shown in logarithmic scale, and significance is tested with the Mann–Whitney U test; the significance is listed in Table S6. PhACs (B and D): AZM—azithromycin, TIM—tilmicosin, GLC—glibenclamide, TIB—thiabendazole, CAZ—carbamazepine, KPF—ketoprofen, NPX—naproxen, SAA—salicylic acid. EDCs (B and D): 1HB—1*H*-benzotriazole, CFN—caffeine, TBEP—tris(2-butoxyethyl)phosphate, TCPP—tris(1-chloro-2-propyl)phosphate, TCEP—tris(2-carboxyethyl)phosphine, PRG—progesterone, BPA—bisphenol A, E3–estriol, TCL—triclosan, MPB—methylparaben, EPB—ethylparaben, PBB—propylparaben.

Odonata, the hemimetabolous insect order (i.e., with incomplete metamorphosis), generally had higher concentrations of ECs than the holometabolous Trichoptera (i.e., having complete metamorphosis) (Figure 3). Additionally, particular life stages showed opposing trends in concentrations between these two orders. In Odonata, for total concentrations of ECs and EDCs and eight individual compounds, significantly higher values were recorded in aquatic larvae than in terrestrial adults (Figure 3A and B). Only three compounds had significantly higher concentrations in adults, and nine showed no difference between two life stages (Figure 3B, Mann–Whitney U test; Table S6). In Trichoptera, the trend was the opposite; for EDCs totals and 11 individual compounds, values were significantly higher in terrestrial adults (Figure 3 C and D). Only one compound measured significantly higher concentrations in aquatic larvae, and eight showed no difference between the two life stages (Figure 3D, M–W U test; Table S6).

Average total concentrations of ECs and PhACs were significantly lower in riparian spiders than observed in aquatic insects (for both aquatic and terrestrial stages; M–W U test; ECs totals *U* = 124, *p* = 0.0005; PhACs totals *U* = 137, *p* = 0.001). Some of the compounds measured in insects were not detected in spiders (TCEP, progesterone, triclosan, Table S5), and *vice versa*, the antibiotic sulfadimethoxine was only measured in riparian spiders at two sites. The remaining compounds did not show significant differences in concentrations between spiders and insects (M–W U test; Table S6), which is in line with observations from some of the Australian rivers where similar concentrations of PhACs were measured in aquatic insect larvae and riparian spiders.^18^

### Compound Specificity and Fate of ECs in Food Webs

In order to infer patterns of ECs transfer through food webs and through the ATHL, a data set with a simplified food web was compiled, including three aquatic compartments: water, biofilm, and Trichoptera larvae (aquatic prey; grazers or grazers and shredders), and two terrestrial: adult Trichoptera (terrestrial prey) and terrestrial predators (riparian spiders or adult dragonflies). Respecting the patterns of mean concentrations of ECs in each compartment, and the bioamplification factors (BAmF) and biomagnification factors (BMF) of two different trends were identified.

**Figure 4:**
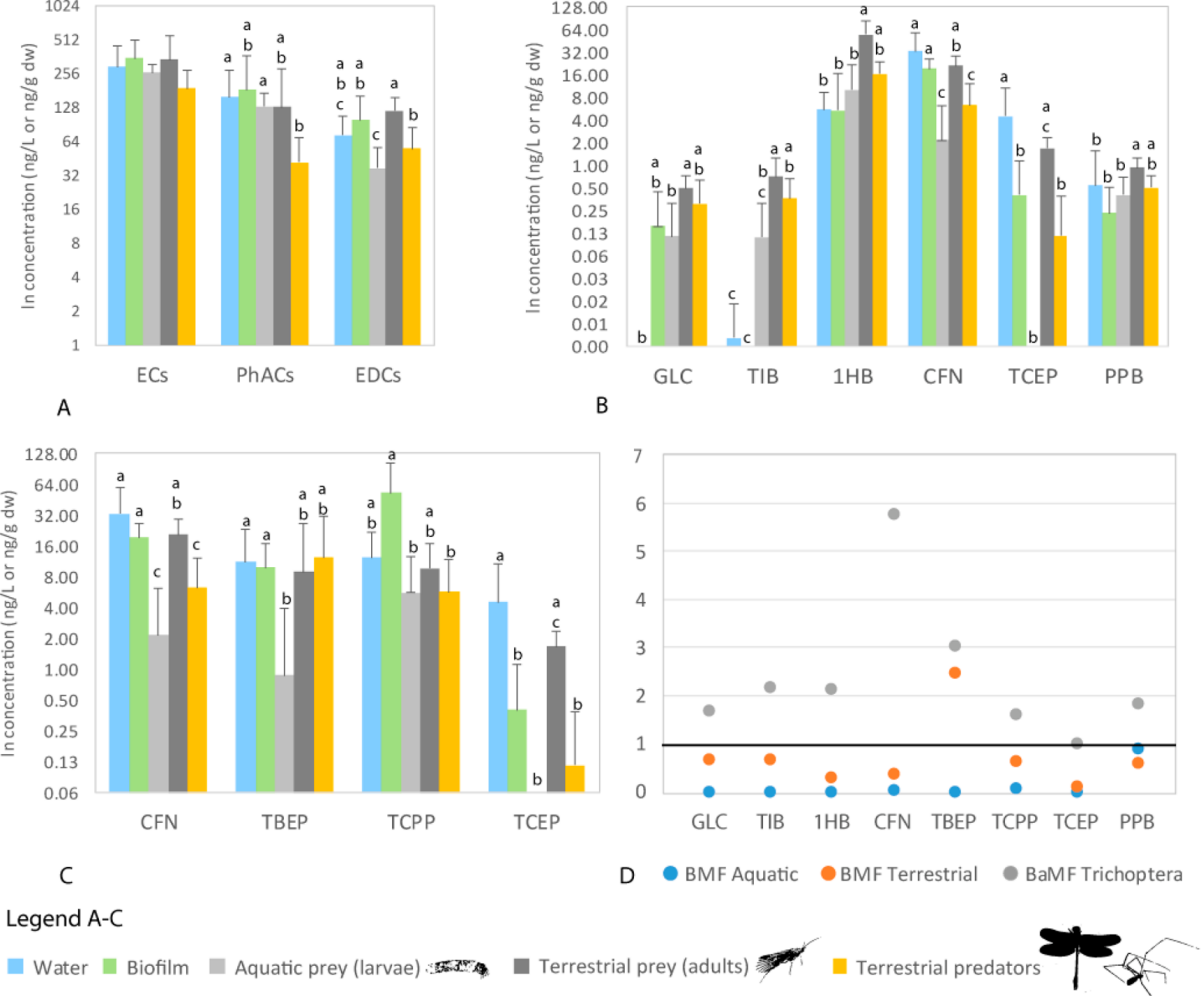
Bioamplification and trophic dilution of pharmaceuticals (PhACs) and endocrine disrupting compounds (EDCs) in the food webs connecting aquatic and terrestrial ecosystems at three sites in NW Croatia. Bioamplification is observed for total EDCs concentration (A) and six compounds (B): glibenclamide (GLC), thiabendazole (TIB), 1*H*-benzotriazole (1HB), caffeine (CFN), tris(2-carboxyethyl)phosphine (TCEP), and propylparaben (PBB) when concentration in adult Trichoptera significantly increases compared to larvae and BAmFs > 1 (D). Trophic dilution in the aquatic part of the food web is observed when the concentration significantly decreases from the biofilm to Trichoptera larvae and BMFs < 1, as observed for total EDCs (A) and three compounds: caffeine (CFN), tris(2-carboxyethyl)phosphine (TCEP), and tris(1-chloro-2-propyl)phosphate (TCPP) (C and D). Trophic dilution in the terrestrial part of the food web is observed when concentration significantly decreases from adult Trichoptera to terrestrial predators and BMFs < 1, as observed for total EDCs (A) and two compounds: caffeine (CFN) and tris(2-carboxyethyl)phosphine (TCEP) (C and D). The significance of the Kruskal–Wallis ANOVA, post hoc tests, and multiple comparisons is listed in Table S7. Biomagnification factors for the aquatic part of the food web (BMF aquatic, D) are calculated as the ratio of the mean concentration of ECs in Trichoptera larvae to the mean concentration of ECs in biofilm, whereas for terrestrial part of the food web (BMF terrestrial, D) as the ratio of the mean concentration of ECs in terrestrial predators to the mean concentration of EC in adult Trichoptera. The bioaplification factor (BaMF Trichoptera, D) was calculated as the ratio of the mean concentration of ECs in adult Trichoptera to the mean concentration of ECs in Trichoptera larvae.

Trend I bioamplification of ECs through the ATHL is observed with significantly increasing concentration in adult Trichoptera, and BAmFs > 1 were identified. Trend I was observed for the total EDCs concentration (Figure 4A) and for six individual compounds: glibenclamide, thiabendazole, 1*H*-benzotriazole, caffeine, TCEP, and propylparaben (Figure 4B and D). This trend may result from the higher body mass loss and low excretion rate of ECs during the metamorphosis of Trichoptera.

Trend II trophic dilution of ECs in either the aquatic or terrestrial part of the food web is observed when the concentration significantly decreases from higher to lower trophic levels and BMFs < 1. For TBEP and TCPP this was observed only in the the aquatic part of the food web (concentrations were significantly lower in grazing Trichoptera larvae than in biofilm samples; Figure 4C and D). On the contrary, for TCEP it was observed only in the terrestrial part; i.e., the concentrations in riparian predators were significantly lower than in adult Trichoptera (Figure 4C). Caffeine was the only compound showing this pattern in both aquatic and terrestrial parts of the food web (Figure 4B and D).

For the remaining compounds, no significant differences between biotic compartments and ecosystems coupled with corresponding patterns of BMFs and BAmFs could be identified (Figure S2A and B). For some of these compounds, BAmFs and/or BMFs > 1 indicate the potential for either bioamplification (e.g., azithromycin), biomagnification (e.g., ketoprofen), or both (e.g., bisphenol A, TBEP); however, a more comprehensive data set is necessary to evaluate these patterns (Figure S2B).

## Discussion

The majority of studies investigating the ecological impacts of ECs have mainly focused on aquatic environments without considering aquatic–terrestrial ecosystem linkages. Only one recent study suggested the possibility of trophic transfer through the aquatic–terrestrial habitat linkage by detecting PhACs in riparian spiders.^18^ Thus, by showing that terrestrial adult insects accumulated PhACs and EDCs in their bodies due to exposure in aquatic larval stages, the current study represents, to our knowledge, the first direct evidence of aquatic–terrestrial ecosystems transfer of these ECs.

Bioaccumulation can occur through bioconcentration and dietary accumulation, as well as bioamplification processes.^31,36^ In the current study, larvae of two different insect orders (Odonata and Trichoptera) bioaccumulated approximately 50% of the PhACs and EDCs present in the water (i.e., 50% of ECs quantified from *in situ* water samples were also quantified in insects) as the result of bioconcentration (e.g., ibuprofen) and/or dietary accumulation (e.g., diphenhydramine). This study shows that Trichoptera, in line with their lower trophic position in food webs (shredders and grazers), generally had lower concentrations of ECs than predatory Odonata, thus supporting the classical biomagnification scenario. Metamorphosis as a physiological process which precedes emergence to the terrestrial ecosystem mediates bioaccumulation.^17^ Bioamplification is defined as the process by which an organism loses body weight and chemical partitioning capacity faster than it can eliminate contaminants.^31^ It usually occurs during specific life history stages (such as insect metamorphosis) that represent major bioenergetic bottlenecks.^31^ In aquatic insects, it has been observed in persistent organic pollutants, i.e., PCBs in mayflies (Ephemeroptera^31^), whereas metals and polycyclic aromatic hydrocarbons have been shown to decrease during insect metamorphosis due to excretion.^17,37^ In Trichoptera, ECs exhibited bioamplification, concentrating in adult stages during metamorphosis. Odonata exhibited the opposite trend, where total concentrations of particular groups of compounds and about half of individual compounds had higher values in aquatic larvae compared to their terrestrial adults. As Odonata remain predators in their adult stages, they have been shown to gain a considerable amount of body mass as adults;^16^ thus, feeding on terrestrial prey with lower concentrations of ECs is reflected in their decreased body burden in their adult terrestrial stages. Although heavily constrained (only three compounds), the observed nonsignificant relationship between physicochemical descriptors and BAF values contributes to recent observations that ECs bioconcentration and bioaccumulation are not easily predicted by simple physicochemical descriptors.^38,39^ It was suggested that traditionally used log *K*_ow_ values do not predict the bioaccumulation potential of ionized compounds well.^38,39^ Also, it was found that ionizable compounds with a log *D* < 1 did not follow a linear relationship with BCF,^39^ consistent with our result where two compounds have log *D* < 1. Moreover, some studies do not even find a clear trend at higher log *D* values,^40^ altogether suggesting deviation from previous models mainly established on persistent organic pollutants. When examining bioaccumulation patterns of PhACs and EDCs at a finer taxonomic resolution, significant differences were observed in the experiment between two Trichoptera taxa of the same family, implying that major uptake routes and bioaccumulation patterns of ECs in aquatic invertebrates may vary at the genus and species level due to differences in life history traits (i.e., feeding behavior). Hence, this observation increases a taxonomical resolution of bioaccumulation of PhACs in aquatic invertebrates to the genus level.

The current study provides important insights regarding the fate and behavior of individual PhACs and EDCs at the aquatic–terrestrial ecosystem boundary. The macrolide antibiotics, azithromycin and tilmicosin, were present in water and all biotic compartments, as well as at all sites, reflecting their wide use and input into the environment. On the contrary, the antibiotic sulfadimethoxine, one of the sulfonamides extensively used in veterinary medicine,^41^ was only measured in riparian spiders, indicating a potentially different source of exposure or a more complex environmental fate. The wide presence of such antibiotics, listed on the EU watch list,^42^ is particularly worrying considering that their subsequent occurrence in the environment is one of the factors contributing to the development of antibiotic resistance.^41^ Ketoprofen (a NSAID) and thiabendazole (an antihelmithic drug) were the only ECs with significantly higher concentrations in adult stages in both insect orders, Odonata and Trichoptera, implying high bioamplification potential irrespective of metamorphosis type. Both of these compounds have low estimated bioconcentration potential in aquatic biota (e.g., fish^43^); however, there are no data on their bioamplification potential. This is of particular concern since ketoprofen has been shown to pose a serious threat to birds (i.e., vultures^44^), and thiabendazole was one of the compounds most frequently detected in bats in the northeastern U.S.^45^ Similarly, 1*H*-benzotriazole also shows potential for bioamplification in emerging insects, as concentrations measured in adult Trichoptera were overall the highest mean concentrations measured *in situ* in the current study. This prompts further investigation, as the toxic effects and/or modification of endocrine activity has been observed in both aquatic and terrestrial organisms.^46^ Trophic dilution observed in the food webs for the majority of ECs, such as the organophosphate flame retardants (OPFRs, e.g., TBEP, TCPP), is in line with the low estimated bioconcentration potential of these compounds in aquatic food webs.^47^ However, studies addressing the environmental fate and toxicity of OPFRs show that different compounds show contrasting patterns.^48^ Hence, the potential for bioamplification in adult Trichoptera (e.g., TCEP) suggests an important transport route of certain OPFRs from aquatic to terrestrial food webs, supplementing previous data on concentrations in various bird taxa.^49^ Therefore, the trophic exposure of riparian predators to ECs showing high bioamplification potential merits further investigation.

Holometabolous aquatic insect orders, such as Diptera and Trichoptera, can constitute a substantial part of benthic production being exported to riparian predators, in which 25–100% of the energy or carbon comes from emerging aquatic insects.^16^ Even the relatively large Odonata are an important transmitting trophic link between small insects, which they feed on, and larger terrestrial predators, such as birds^50−52^ and bats.^53,54^ Hence, these vertebrate predators consume the largest amount of emerging aquatic insects (e.g., up to 90% of daily emergence^16^). Taking into account the latter, coupled with the estimated food consumption of insectivorous birds during the breeding season (e.g., *Parus major*, 3.145 g of dry food matter per day^55^), gives an estimated potential daily intake of ca. 378 ng of total EDCs and 408 ng of total PhACs measured in the current study by the riparian birds. For individual compounds, this would give an estimate of ca. 55–62 ng/day for ketoprofen, caffeine, and salicylic acid, to maximally ca. 157 ng/day of 1*H*-benzotriazole. Similarly, if estimates for the consumption of insectivorous bats are applied (e.g., for *Pipistrellus pipistrellus* between 0.68 and 1.35 g of insects from a single location are consumed per night^47^), an average of daily intake up to 175.5 ng of total EDCs and 162 ng of total PhACs can be expected. Dominant Trichoptera species collected in the current study were recorded to emerge in high abundance for at least four months (i.e., spring–autumn; *Silo nigricornis*(56)), with an average population density of larvae around 590 individuals m^–2^ between April and November. Predatory birds and bats, such as *P. major* and *P. pipistrellus*, could therefore consume 44.25 and 67.5 ng of ECs per g of their body weight on a daily basis over the course of several months, respectively. Each individual PhAC consumed by these riparian predators is therefore well below the equivalent of 1% of average human daily dose.^57^ However, it remains open to see if there are any interactive effects of PhACs in the mixture and whether the equivalent of the human daily dose is applicable to nontargeted organisms. On the other hand, levels at which some of the EDCs like estriol and progesterone are consumed by these predators would be from 480% to 5000% equivalent of the physiological daily level in humans, respectively.^58,59^ It is speculated that if ECs are transported through the ATHL they could potentially affect not only the physiology of individuals but also the population dynamics of riparian-dependent terrestrial predators.^60^ For instance, in European starlings feeding on invertebrates from within filter beds of sewage treatment works, adverse population level effects have been attributed to the trophic exposure of EDCs, seen through reduced immunocompetence and growth, modifications in song behavior, and neural development.^61^ Additionally, considerable differences in ECs transport are expected at a spatial scale, as the lateral extent of the subsidy was shown to decline considerably with distance from the stream channel in riparian spiders.^62^ Hence, considering the immature understanding of ecological impacts, as well as the interactive effects of mixtures of ECs actually found in the environment, our results highlight the need for further multidisciplinary research of ECs propagating across ecosystem boundaries.

## Supporting Information Available

Details on UPLC separation; Spearman’s rank correlation between log BAF values and physicochemical descriptors of ECs used in experimental treatment (Figure S1); concentrations, biomagnification, and bioamplification factors (Figure S2); additional data and results and full names of compounds and abbreviations (Tables S1–S7) (pdf)

## Notes

All data needed to evaluate the conclusions of the study are present in the paper and/or the Supporting Information. Additional data related to the paper may be requested from the authors.
