# Supplementary material for: Aquatic Insects Transfer Pharmaceuticals and Endocrine Disruptors from Aquatic to Terrestrial Ecosystems
Source: Environ Sci Technol. 2021 Mar 2;55(6):3736–46. doi: 10.1021/acs.est.0c07609 (PMC8031366; doi:10.1021/acs.est.0c07609)
Supplement: Supplementary file 1 — es0c07609_si_001.pdf [file es0c07609_si_001.pdf]

Supporting Information: Methods

UPLC separation:

Target analysis was performed using an ultra-performance liquid chromatography (UPLC) system (Waters Milford, MA, USA) coupled to a hybrid quadrupole linear ion trap mass spectrometer Qtrap 5500 (Applied Biosystems, Foster City, CA, USA). Following chromatographic columns were used: PhACs positive ion mode (PIM) and EDCs PIM - Waters Acquity HSS T3 (50 mm × 2.1 mm i.d., 1.8 µm particle size), PhACs negative ion mode (NIM) and EDCs NIM – Waters Acquity BEH C18 (50 mm × 2.1 mm i.d., 1.7 µm particle size).

For the separation of PhACs in PIM methanol (solvent A) and 10 mM ammonium formate at pH 3.2 (solvent B) at the 0.5 ml/min flow rate were used. The gradient elution was: 0 - 4.5 min, 5 – 95 % A; 4.5 – 6 min, 100 % A; 6 - 6.7 min, 5 % A.

For the separation of PhACs in NIM acetonitrile (solvent A) and 10 mM ammonium acetate at pH 8 (solvent B) at the 0.6 ml/min flow rate were used. The gradient elution was: 0 - 1.5 min, 5 – 60 % A; 1.5 – 2 min, 60-100 % A; 2 - 3 min, 100 % A; 3.2 -3.7 min, 5 % A.

For the separation of EDCs in PIM methanol (solvent A) and 10 mM ammonium formate at pH 3.2 (solvent B) at the 0.4 ml/min flow rate were used. The gradient elution was: 0–3 min, 30 – 100 % A; 3 – 4.75 min, 100 % A; 4.75 – 5.75 min, 100-30 % A; 5.75–7 min, 30 % A.

For the separation of EDCs in NIM methanol (solvent A) and water at pH 9 solvent (B) at the flow rate 0.4 ml/min were used. The gradient elution was: 0 – 4 min, 30–100 % A; 4–5 min, 100 % A; 5–6 min, 100-30 % A; 6–7.5 min, 30 % A.

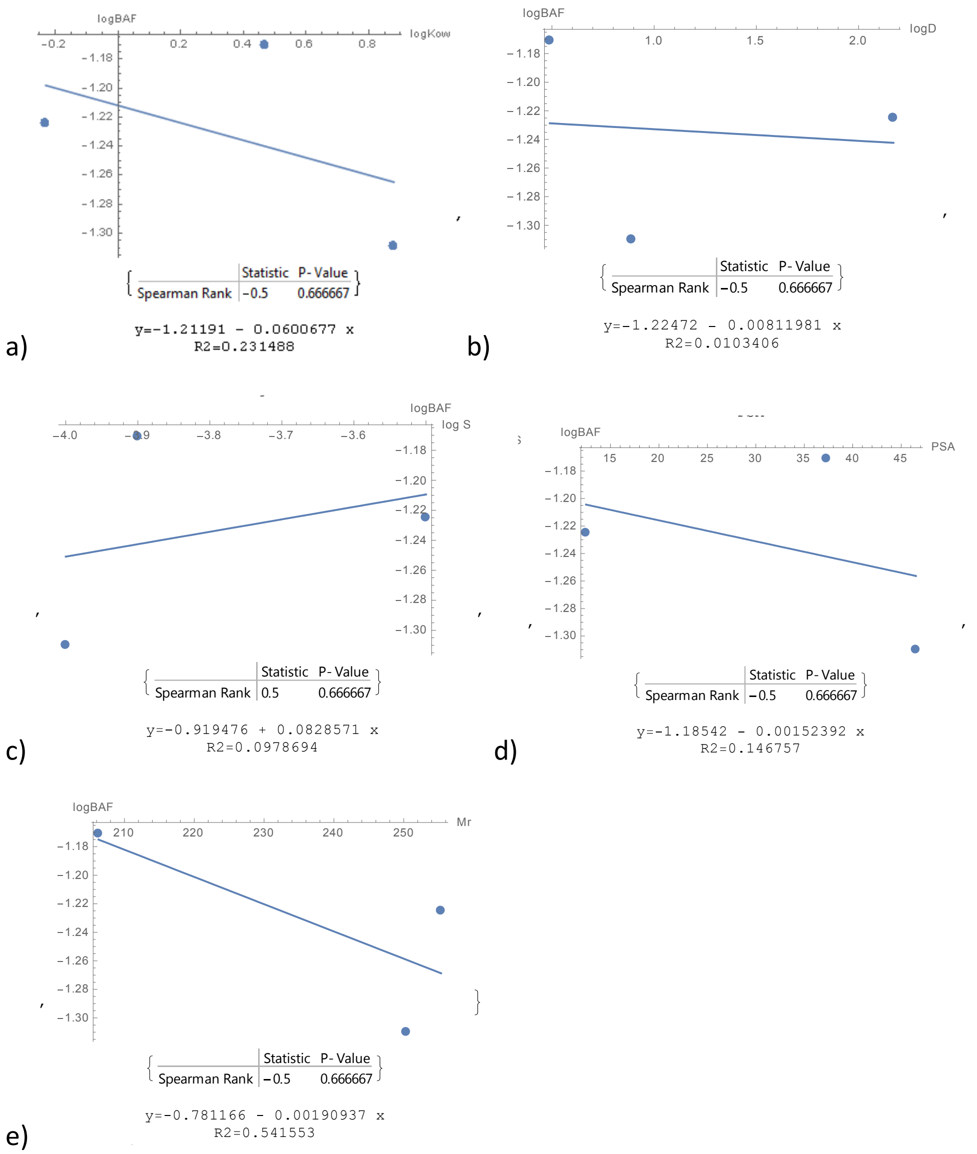

Figure S1. Spearman's rank correlation (and simple linear regression model) between log BAF values (for *D. croaticus* larvae) and physicochemical descriptors of ECs used in experimental treatment: a) the octanol–water partition coefficient (log K_{ow}), b) octanol–water distribution coefficient (log *D*), c) aqueous solubility (log *S*), d) polar surface area (PSA) and e) relative molecular mass (*M*r).

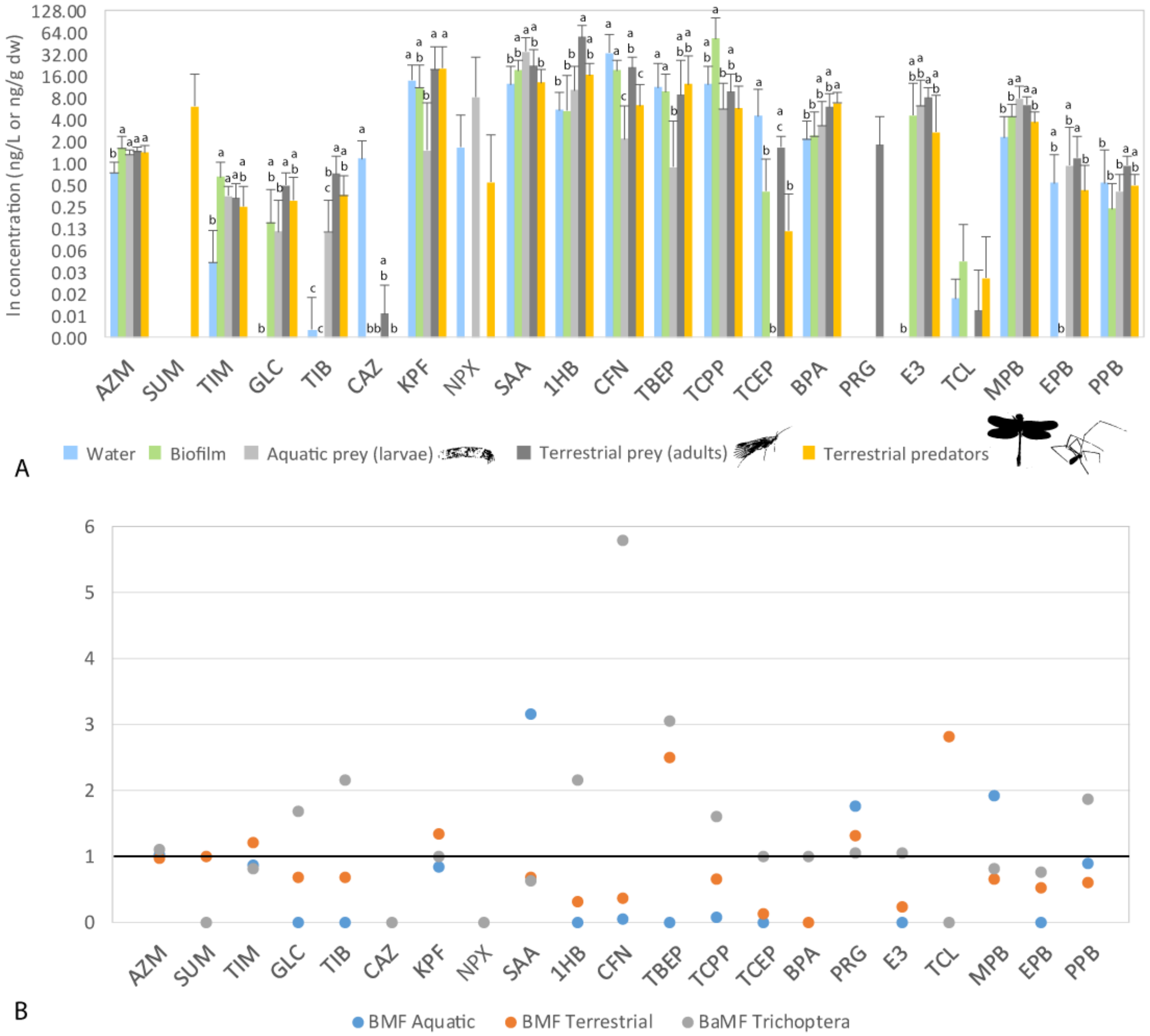

Figure S2. Concentrations (**A**) and biomagnification (BAFs; **B**) and bioamplification factors (BaMFs; **B**) of pharmaceuticals (PhACs) and endocrine disrupting compounds (EDCs) in the food webs connecting aquatic and terrestrial ecosystems at 3 sites in NW Croatia. Significance of the Kruskal-Wallis ANOVA, post hoc tests and Multiple comparisons is listed in Table S7. Biomagnification factors for aquatic part of the food web (BMF Aquatic, **B**) are calculated as the ratio of mean concentration of ECs in caddisfly larvae to mean concentration of ECs in biofilm, whereas for terrestrial part of the food web (BMF Terrestrial, **B**) as the ratio of mean concentration of ECs in terrestrial predators to mean concentration of EC in adult caddisflies. Bioaplification factor (BaMF Trichoptera, **B**) was calculated as the ratio of mean concentration of ECs in adult caddisflies to mean concentration of ECs in caddisfly larvae. Names and abbreviations of compounds are listed in Table S5.

Table S1. Information on 5 sampling sites in NW Croatia were *in situ* sampling was conducted in 2018. and on taxa collected.

Sampling site	Habitat	Coordinates	Sampling dates	Pollution impacts	Type of samples (taxa collected)
Krapina River - Kupjenovo (KK)	medium sized lowland river (hydromorphologically altered)	N45.934457 E15.818039	29.05.2018. & 18.06.2018.	communal and hospital untreated effluents, agricultural runoff	water, biofilm<br>Odonata larvae (*Gomphus* sp., *Onychogomphus* sp., *Orthetrum* sp., *Calopteryx* sp., *Platycnemis pennipes*)<br>Odonata adults (*Gomphus vulgatissimus*, *Onychogomphus forcipatus*, *Orthetrum albistylum*, *Calopteryx splendens*, *Platycnemis pennipes*)<br>Trichoptera larvae (*Hydropsyche contubernalis*)
Krapina River - Zaprešić (KZ)	medium sized lowland river (hydromorphologically altered)	N45.834771 E15.822810	29.05.2018. & 18.06.2018.	communal and hospital untreated effluents, agricultural runoff	water, biofilm<br>Odonata larvae (*Calopteryx* sp., *Platycnemis pennipes*)<br>Odonata adults (*Calopteryx splendens*, *Platycnemis pennipes*)
Bistrec stream (BI)	lowland stream (hydromorphologically altered)	N46.338223 E16.808941	23.04.2018. & 22.05.2018.	communal untreated effluents, agricultural runoff	water, biofilm<br>Odonata larvae (*Gomphus* sp., *Calopteryx* sp.)<br>Odonata adults (*Gomphus* sp., *Calopteryx* sp.)<br>Trichoptera larvae (*Anabolia* sp.)<br>Trichoptera adults (*Notidobia* sp.)
Čakovec drainage ditch (VZ)	HPP drainage ditch (habitat similar to large lowland stream with gravel and cobbles)	N46.317881 E16.435946	23.04.2018. & 22.05.2018.	effluent of WWTP	water, biofilm<br>Trichoptera larvae (Limnephilidae Gen.sp.)<br>Trichoptera adults (*Notidobia ciliaris*)<br>riparian spiders (Araneaidae)
Dubrava drainage ditch (PR)	HPP drainage ditch (habitat similar to large lowland stream with gravel and cobbles)	N46.323477 E16.634145	23.04.2018. & 22.05.2018.	untreated communal effluent, agricultural runoff	water, biofilm<br>Trichoptera larvae (*Silo nigricornis* , Limnephilidae Gen.sp., *Hydropsyche* sp.)<br>Trichoptera adults (*Silo nigricornis*)<br>riparian spiders (Araneaidae)

Physico-chemical water parameters*

Sampling site	Water temperature (°C)			pH value			Conductivity (µS/cm)			Alcalinity (mg $CaCO_3$/l)			Oxygen concentration (mg O_2/l)		
	average	min	max	average	min	max	average	min	max	average	min	max	average	min	max
KK	13,42	2,80	23,90	8,01	7,63	8,32	679,25	521,00	765,00	304,91	254,00	328,00	7,89	1,70	13,40
KZ	15,40	5,10	26,10	8,03	8,00	8,10	587,14	460,00	636,00	311,83	270,40	347,60	9,85	8,10	11,80
BI	15,18	8,10	22,20	8,03	7,90	8,20	725,33	694,00	756,00	186,75	130,00	240,00	10,25	8,70	11,60
VZ	11,75	1,20	16,20	8,12	7,92	8,27	335,75	316,00	347,00	165,50	154,00	179,00	7,58	5,99	8,73
PR	11,48	1,80	15,20	7,98	7,91	8,12	344,00	328,00	368,00	159,75	143,00	172,00	5,81	4,89	6,64

Sampling site	Oxygen saturation (%)			BPK_5 (mgO_2/l)			KPK-Mn (mgO_2/l)			Amonium (mgN/l)		
	average	min	max	average	min	max	average	min	max	average	min	max
KK	72,49	16,60	113,90	2,83	1,80	4,40	4,22	2,70	8,80	0,70	0,23	1,14
KZ	97,15	88,24	120,10	3,07	1,93	3,00	3,70	2,30	5,40	0,13	0,05	0,30
BI	101,30	90,60	110,60	3,32	3,00	4,00	2,07	1,50	2,60	0,06	0,21	3,87
VZ	77,38	55,70	89,70	0,65	< 0.5	0,80	1,26	0,48	2,29	< 0.03	0,05	0,20
PR	58,08	45,20	67,30	< 0.5			1,29	0,64	1,94	< 0.03		

Sampling site	Nitrates (mgN/l)			Total N (mg N/l)			Ortophosphates (mgP/l)			Total P (mg P/l)		
	average	min	max	average	min	max	average	min	max	average	min	max
KK	1,10	0,29	1,73	2,10	1,51	3,15	0,09	0,02	0,21	0,28	0,12	0,52
KZ	1,22	0,10	1,34	1,75	1,57	1,90	0,04	0,02	0,06	0,14	0,10	0,16
BI	3,36	4,19	4,30	3,70	4,80	0,12	0,12	0,12	0,12	<0.3		
VZ	< 0.5			0,70	< 0.5	0,80	0,05	< 0.01	0,09	0,10	0,07	0,12
PR	< 0.5			0,63	< 0.5	0,80	0,02	< 0.01	0,06	0,04	0,01	0,10

*for KK, KZ and BI yearly values obtained from Hrvatske vode; for VZ and PR from Report on quality of surface and subterranean waters of HPP Čakovec and Dubrava, Biointitut Čakovec, 2018.

Table S2. List of 6 compounds screened for in the microcosm experiment (4 PhACs and 2 EDCs) and 143 compounds screened for in the in situ samples (119 PhACs 24 EDCs). For diphenhydramine, gemfibrozil and ibuprofen physicochemical descriptors are listed; the octanol–water partition coefficient (log K_{ow}), octanol–water distribution coefficient (log *D*), aqueous solubility (log *S*), polar surface area (PSA) and relative molecular mass (Mr).

List of compounds screened in the mesocosm experiment samples (4 PhACs and 2 EDCs)

	log K_{ow}	log *D*	log *S*	PSA	Mr
Diphenhydramine	3.27	2.17	-3.5	12.47	255.35
Gemfibrozil	4.38	0.89	-4	46.53	250.38
Ibuprofen	3.97	0.49	-3.9	37.3	206.285
Diclofenac					
Methylparaben					
Tri(2-butoxyethanol) phosphate, TBEP					

List of compounds screened in the in situ samples (24 EDCs and 119 PhACs)

**EDC**		
1H-benzotriazole	Estradiol, E2	Octylphenol
Benzylparaben	Estradiol 17?-glucuroni[illegible]	Progesterone
Bisphenol A, BPA	Estriol, E3	Propylparaben
Caffeine	Ethinylestradiol, EE2	Tri(2-butoxyethanol) phosphate, TBEP
Diethylstibesterol	Ethylparaben	tris(2-carboxyethyl)phosphine, TCEP
Estrone, E1	Levonorgestrel	Tris(1-chloro-2-propanyl) phosphate, TCPP
Estrone 3-glucuronide, E1-3G	Methylparaben	Tolyltriazole
Estrone sulfate, E1-3S	Nonylphenol	Triclosan
**PhACs**		
1oh-ibuprofen	Doxycycline	Oxytetracyclin
2-oh carbamazepine	Enrofloxacin	Paroxetine
2OH ibuprofen	Epoxy Carbamazepine	Penicillinv
3oh acetaminophen	Erythromycin	Phenazone
4oh diclofenac	Famotidine	Pipemidic acid
5oh diclofenac	Florfenicol	Piroxicam
Acetaminophen	Flubendazole	Pravastatin
Acridone	Flunixin	Propanolol
Albendazole	Fluoxetine	Propylphenazone
Amlodipine	Fluvastatin	Ranitidine
Amoxicillin	Furosemide	Ronidazole
Ampicillin	Gemfibrozil	Roxithromycin
Atenelol	Glibenclamide	Salbutamol
Atorvastatin	Hydrochlorothiazide	Salicylic acid
Azaperol	Ibuprofen	Sertraline
Azaperone	Indomethacine	Sildenafil
Azithromycin	Irbesartan	Sotalol
Bezafibrate	Ketoprofen	Spiramycin
Carazolol	Levamisol	Sulfadiazine
Carbamazepine	Lincomycin	Sulfadimethoxine
Carboxy ibuprofen	Loratadine	Sulfamethazine
Cefalexin	Lorazepam	Sulfamethoxazole
Ceftiofur	Losartan	Sulfapyridine
Chloramphenicol	Marbofloxacin	Sulfisoxazole
Chlortetracycline	Meloxicam	Tamsulosin
Cimitedine	Metoprolol	Tenoxicam
Ciproflox	Metoprololcid	Tetracyclin
Ciprofloxacin	Metronidazole	Thiabendazole
Citalopram	N-Acetyl-Sulfamethoxazole	Tiamulin
Clarithromycin	Nadolol	Tiamullinc
Clindamycin	Naproxen	Tilmicosin
Clopridogrel	N-Desmethyl-venlafaxine	Torasemide
Codeine	Norfloxacin	Trazodone
Desloratadine	Norverapamil	Trimethoprim
Dexamethasone	O-Desmetyl-venlafaxine	Tylosin
Diclofenac	Ofloxacin	Valsartan
Diclofenac-acyl-gluc	OH-metronidazole	Venlafaxine
Dilitiazem	Olanzapine	Verapamil
Dimetridazole	Oxolinicacid	Warfarin
	Oxycodone	Xylazine

Table S3. Results of nonparametric tests (Wald and ANOVA tests & Multiple comparisons) showing significance of changes in mean concentrations of pharmaceuticals (PhACs) and endocrine disrupting compounds (EDCs) in tissues of two caddisfly taxa in the microcosm experiment.
In all compounds except methylparaben multiple comparisons tests were conducted only in experimental treatments, in methylparaben both were tested separately, control and experimental treatments. Significant *p* values in red.

Caddisflies

Diphenhydramine (DPA)

	**Wald test**			**ANOVA test**		
	Statistic	df	p-value	Statistic	df	p-value
species	4,506	1	0,0338	4,506	1,000	0,0338
treatment	441,197	1	< 0.0001	441,197	1,000	< 0.0001
time	103,375	4	< 0.0001	19,099	2,409	< 0.0001
species:treatment	3,196	1	0,0738	3,196	1,000	0,0738
species:time	113,342	4	< 0.0001	9,629	2,409	< 0.0001
treatment:time	142,561	4	< 0.0001	26,428	2,409	< 0.0001
species:treatment:time	61,882	4	< 0.0001	3,025	2,409	0,0390

Multiple comparisons	**Wald test & ANOVA test**			Multiple comparisons	**Wald test & ANOVA test**		
Drusus croaticus	Statistic	df	p-value	***Potamophylax* sp.**	Statistic	df	p-value
Time 0-21	9,00	1	0,0027	Time 0-21	27,00	1	< 0.0001
Time 0-35	9,00	1	0,0027	Time 0-35	6,75	1	0,0094
Time 0-65	6,75	1	0,0094	Time 0-65	27,00	1	< 0.0001
Time 0-65 (IM)	27,00	1	< 0.0001	Time 0-65 (IM)	27,00	1	< 0.0001
Time 21-35	0,33	1	0,5637	Time 21-35	1,75	1	0,1859
Time 21-65	27,00	1	< 0.0001	Time 21-65	0,81	1	0,3692
Time 21-65 (IM)	0,23	1	0,6310	Time 21-65 (IM)	9,00	1	0,0027
Time 35-65	7,00	1	0,0082	Time 35-65	9,00	1	0,0027
Time 35-65 (IM)	0,08	1	0,7815	Time 35-65 (IM)	27,00	1	< 0.0001
Time 65-65 (IM)	9,00	1	0,0027	Time 65-65 (IM)	0,08	1	0,7815

Gemfibrozil (GFZ)

	**Wald test**			**ANOVA test**		
	Statistic	df	p-value	Statistic	df	p-value
species	20,090	1	< 0.0001	20,090	1,000	< 0.0001
treatment	41,565	1	< 0.0001	41,565	1,000	0,0001
time	1032,384	4	< 0.0001	16,887	1,013	< 0.0001
species:treatment	20,090	1	< 0.0001	20,090	1,000	< 0.0001
species:time	1034,419	4	< 0.0001	8,044	1,013	0,0044
treatment:time	1032,384	4	< 0.0001	16,887	1,013	< 0.0001
species:treatment:time	1034,419	4	< 0.0001	8,044	1,013	0,0044

Multiple comparisons	**Wald test & ANOVA test**			Multiple comparisons			
Drusus croaticus	Statistic	df	p-value	***Potamophylax* sp.**	Statistic	df	p-value
Time 0-21				Time 0-21			
Time 0-35	27,00	1	< 0.0001	Time 0-35	1,00	1	0,3173
Time 0-65				Time 0-65			
Time 0-65 (IM)	27,00	1	< 0.0001	Time 0-65 (IM)			
Time 21-35	27,00	1	< 0.0001	Time 21-35			
Time 21-65	27,00	1	< 0.0001	Time 21-65			
Time 21-65 (IM)				Time 21-65 (IM)			
Time 35-65				Time 35-65			
Time 35-65 (IM)	49,00	1	< 0.0001	Time 35-65 (IM)			
Time 65-65 (IM)	27,00	1	< 0.0001	Time 65-65 (IM)			

Ibuprofen (IPF)

	**Wald test**			**ANOVA test**		
	Statistic	df	p-value	Statistic	df	p-value
species	17,729	1	< 0.0001	17,729	1	< 0.0001
treatment	17,729	1	< 0.0001	17,729	1	< 0.0001
time	0,039	4	0,9998	9,702	1	0,0018
species:treatment	17,729	1	< 0.0001	17,729	1	< 0.0001
species:time	0,039	4	0,9998	9,702	1	0,0018
treatment:time	0,039	4	0,9998	9,702	1	0,0018
species:treatment:time	0,039	4	0,9998	9,702	1	0,0018

Multiple comparisons	**Wald test & ANOVA test**		
Drusus croaticus	Statistic	df	p-value
Time 0-21	1,00	1	0,3173
Time 0-35			
Time 0-65			
Time 0-65 (IM)	1,00	1	0,3173
Time 21-35			
Time 21-65			
Time 21-65 (IM)			
Time 35-65			
Time 35-65 (IM)	1,00	1	0,3173
Time 65-65 (IM)			

Methylparaben (MPB)

	**Wald test**			**ANOVA test**		
	Statistic	df	p-value	Statistic	df	p-value
species	314,405	1	< 0.0001	314,405	1,000	< 0.0001
treatment	7,969	1	0,0048	7,969	1,000	0,0048
time	0,146	4	0,9975	0,062	2,396	0,9613
species:treatment	8,475	1	0,0036	8,475	1,000	0,0036
species:time	136,290	4	< 0.0001	21,307	2,396	< 0.0001
treatment:time	18,605	4	0,0009	1,854	2,396	0,1480
species:treatment:time	29,992	4	< 0.0001	1,232	2,396	0,2946

Multiple comparisons	**Wald test & ANOVA test - EXPERIMENT**			**Wald test & ANOVA test - CONTROL**			Multiple comparisons	**Wald test & ANOVA test - EXPERIMENT**			**Wald test & ANOVA test - CONTROL**		
Drusus croaticus	Statistic	df	p-value	Statistic	df	p-value	***Potamophylax* sp.**	Statistic	df	p-value	Statistic	df	p-value
Time 0-21	0,25	1	0,6171	9,00	1	0,0027	Time 0-21	1,96	1	0,1615	0,04	1	0,8415
Time 0-35	9,00	1	0,0027	27,00	1	< 0.0001	Time 0-35	9,00	1	0,0027	7,00	1	0,0082
Time 0-65	27,00	1	< 0.0001	27,00	1	< 0.0001	Time 0-65	0,89	1	0,3447	27,00	1	< 0.0001
Time 0-65 (IM)	9,00	1	0,0027	27,00	1	< 0.0001	Time 0-65 (IM)	27,00	1	< 0.0001	27,00	1	< 0.0001
Time 21-35	0	0	NA	0,75	1	0,3859	Time 21-35	49,00	1	< 0.0001	0,75	1	0,3865
Time 21-65	27,00	1	< 0.0001	0,43	1	0,5127	Time 21-65	0,02	1	0,8788	27,00	1	< 0.0001
Time 21-65 (IM)	0	0	NA	27,00	1	< 0.0001	Time 21-65 (IM)	27,00	1	< 0.0001	27,00	1	< 0.0001
Time 35-65	27,00	1	< 0.0001	1,00	1	0,3173	Time 35-65	3,06	1	0,0801	0,81	1	0,3692
Time 35-65 (IM)	25,00	1	< 0.0001	1,00	1	0,3173	Time 35-65 (IM)	0,43	1	0,5127	27,00	1	< 0.0001
Time 65-65 (IM)	27,00	1	< 0.0001	1,00	1	0,3173	Time 65-65 (IM)	27,00	1	< 0.0001	27,00	1	< 0.0001

Table S4. Results of nonparametric tests (ANOVA and Wald tests & Multiple comparisons) showing significance of changes in mean concentrations of pharmaceuticals (PhACs) and endocrine disrupting compounds (EDCs) in tissues in moss tissues in the microcosm experiment. In all compounds except methylparaben Multiple comparisons tests were conducted only in experimental treatments, in methylparaben both, control and experimental treatments were tested separately. Significant p values in red.

Diclofenac (DCF)

**Wald test**	Statistic	df	p-value	**ANOVA test**	Statistic	df	p-value
treatment	204,632	1	< 0.0001	treatment	204,632	1,000	< 0.0001
time	159,787	2	< 0.0001	time	126,400	1,545	< 0.0001
treatment:time	159,787	2	< 0.0001	treatment:time	126,400	1,545	< 0.0001

Multiple comparisons	**Wald test & ANOVA test**		
	Statistic	df	p-value
Time 0-21	27,00	1	< 0.0001
Time 0-35	27,00	1	< 0.0001
Time 0-65	27,00	1	< 0.0001
Time 21-35	6,25	1	0,0124
Time 21-65	27,00	1	< 0.0001
Time 35-65	27,00	1	< 0.0001

Diphenhydramine (DPA)

**Wald test**	Statistic	df	p-value	**ANOVA test**	Statistic	df	p-value
treatment	87,397	1	< 0.0001	treatment	87,397	1,000	< 0.0001
time	140,773	3	< 0.0001	time	14,820	1,915	0,5963
treatment:time	21,409	3	86,5869	treatment:time	9,579	1,915	91,6779

Multiple comparisons	**Wald test & ANOVA test**		
	Statistic	df	p-value
Time 0-21	9,00	1	0,0027
Time 0-35	9,00	1	0,0027
Time 0-65	27,00	1	< 0.0001
Time 21-35	27,00	1	< 0.0001
Time 21-65	1,75	1	0,1859
Time 35-65	6,75	1	0,0094

Gemfibrozil (GFZ)

**Wald test**	Statistic	df	p-value	**ANOVA test**	Statistic	df	p-value
treatment	204,632	1	< 0.0001	treatment	204,632	1,000	< 0.0001
time	37,170	2	0,0085	time	16,803	1,189	11,6185
treatment:time	37,170	2	0,0085	treatment:time	16,803	1,189	11,6185

Multiple comparisons	**Wald test & ANOVA test**		
	Statistic	df	p-value
Time 0-21	27,00	1	< 0.0001
Time 0-35	27,00	1	< 0.0001
Time 0-65	27,00	1	< 0.0001
Time 21-35	6,25	1	0,0124
Time 21-65	0,19	1	0,6650
Time 35-65	0,25	1	0,6171

Methylparaben (MPB)

**Wald test**	Statistic	df	p-value	**ANOVA test**	Statistic	df	p-value
treatment	0,022	1	0,8819	treatment	0,022	1,000	0,8819
time	234,474	3	< 0.0001	time	2,855	1,393	0,0767
treatment:time	26,327	3	< 0.0001	treatment:time	0,239	1,393	0,7047

Multiple comparisons	**Wald test & ANOVA test - EXPERIMENT**		
	Statistic	df	p-value
Time 0-21	0,08	1	0,7815
Time 0-35	7,00	1	0,0082
Time 0-65	25,00	1	< 0.0001
Time 21-35	1,96	1	0,1615
Time 21-65	0,75	1	0,3865
Time 35-65	0,75	1	0,3865

Multiple comparisons	**Wald test & ANOVA test - CONTROL**		
	Statistic	df	p-value
Time 0-21	1,00	1	0,3173
Time 0-35	0,81	1	0,3692
Time 0-65	25,00	1	< 0.0001
Time 21-35	0,25	1	0,6171
Time 21-65	25,00	1	< 0.0001
Time 35-65	0,25	1	0,6171

TBEP

**Wald test**	Statistic	df	p-value	**ANOVA test**	Statistic	df	p-value
treatment	26,959	1	0,2078	treatment	26,959	1,000	< 0.0001
time	1762,346	3	< 0.0001	time	13,888	1,746	< 0.0001
treatment:time	1695,993	3	< 0.0001	treatment:time	7,977	1,746	0,0007

Multiple comparisons	**Wald test & ANOVA test**		
	Statistic	df	p-value
Time 0-21	27,00	1	< 0.0001
Time 0-35	27,00	1	< 0.0001
Time 0-65	1,00	1	0,3173
Time 21-35	3,06	1	0,0801
Time 21-65	108,00	1	< 0.0001
Time 35-65	15,43	1	< 0.0001

Table S5. Total number of compounds (pharmaceuticals [PhACs] and endocrine disrupting compounds [EDCs]) recorded at particular data level (N) and mean concentrations (in ng/g of dry weight) measured in the field study conducted at 5 sites in NW Croatia.
Totals of emerging contaminants (ECs), pharmaceuticals (PhACs) and endocrine disrupting compounds (EDCs), s.d. - standard deviation, n.d. - compounds not detected (below detection limit)

Data level	N ECs	Total ECs	Total PhACs	Total EDCs	Azithromycin AZM	Clarithromycin CLM	Cefalexin CFL	Metronidazole MNZ	Sulfadimethoxine SUM	Sulfamethoxazole SMZ	Tilmicosin TIM	Trimethoprim TMP	Clopridogrel CPG	Warfarin WAF	Glibenclamide GLC	Thiabendazole TIB	Azaperol AZL	Metoprolol MEP	Iopromide IOP	Norfluoxetine NOF	Carbamazepine CAZ
					Pharmaceuticals (PhACs)																
**Water**	40	1084,159	827,421	256,738	0,718	1,349	2,012	0,908	n.d.	2,289	0,033	4,569	0,043	0,181	n.d.	0,077	0,031	0,099	20,699	n.d.	3,256
**s.d.**		1342,903	1070,019	272,883	0,345	1,771	3,594	1,427		3,068	0,066	5,865	0,062	0,229		0,148	0,082	0,182	56,704		3,511
**Biofilm**	26	335,846	257,588	78,258	1,138	0,105	n.d.	n.d.	n.d.	n.d.	0,276	n.d.	n.d.	n.d.	0,102	n.d.	0,023	n.d.	n.d.	148,344	0,070
**s.d.**		270,984	279,137	55,728	0,560	0,155					0,365				0,234		0,095			242,074	0,103
**Aquatic insects - total**	20	242,922	117,280	123,656	1,621	n.d.	n.d.	n.d.	n.d.	n.d.	0,366	n.d.	n.d.	n.d.	0,435	0,209	n.d.	n.d.	n.d.	n.d.	0,003
**s.d.**		178,627	109,485	155,517	0,670						0,272				0,393	0,379					0,010
**Aquatic insects - aquatic**	19	292,203	124,144	165,978	1,660	n.d.	n.d.	n.d.	n.d.	n.d.	0,421	n.d.	n.d.	n.d.	0,310	0,036	n.d.	n.d.	n.d.	n.d.	0,004
**s.d.**		204,760	114,275	197,850	0,646						0,240				0,392	0,114					0,011
**Aquatic insects - terrestrial**	18	183,081	108,945	72,265	1,573	n.d.	n.d.	n.d.	n.d.	n.d.	0,299	n.d.	n.d.	n.d.	0,588	0,419	n.d.	n.d.	n.d.	n.d.	0,002
**s.d.**		117,380	104,127	39,984	0,703						0,296				0,341	0,474					0,007
**Odonata total**	20	266,147	107,097	156,952	1,710	n.d.	n.d.	n.d.	n.d.	n.d.	0,387	n.d.	n.d.	n.d.	0,570	0,160	n.d.	n.d.	n.d.	n.d.	0,003
**s.d.**		203,302	107,666	188,099	0,798						0,313				0,398	0,327					0,011
**Odonata aquatic**	18	389,034	114,307	272,414	1,816	n.d.	n.d.	n.d.	n.d.	n.d.	0,497	n.d.	n.d.	n.d.	0,506	n.d.	n.d.	n.d.	n.d.	n.d.	0,007
**s.d.**		228,997	135,297	222,291	0,774						0,261				0,427						0,015
**Odonata terrestrial**	18	158,380	107,175	49,371	1,576	n.d.	n.d.	n.d.	n.d.	n.d.	0,258	n.d.	n.d.	n.d.	0,612	0,338	n.d.	n.d.	n.d.	n.d.	n.d.
**s.d.**		81,873	78,177	18,686	0,856						0,331				0,385	0,410					
**Trichoptera total**	18	203,300	129,077	72,410	1,478	n.d.	n.d.	n.d.	n.d.	n.d.	0,335	n.d.	n.d.	n.d.	0,229	0,294	n.d.	n.d.	n.d.	n.d.	0,003
**s.d.**		125,001	110,454	47,808	0,362						0,191				0,278	0,448					0,008
**Trichoptera aquatic**	16	170,303	131,356	37,287	1,304	n.d.	n.d.	n.d.	n.d.	n.d.	0,356	n.d.	n.d.	n.d.	0,112	0,109	n.d.	n.d.	n.d.	n.d.	n.d.
**s.d.**		53,429	40,507	19,904	0,226						0,121				0,203	0,199					
**Trichoptera terrestrial**	18	251,915	129,787	120,335	1,467	n.d.	n.d.	n.d.	n.d.	n.d.	0,325	n.d.	n.d.	n.d.	0,488	0,705	n.d.	n.d.	n.d.	n.d.	0,008
**s.d.**		167,565	155,710	35,703	0,234						0,207				0,248	0,536					0,012
**Riparian spiders**	18	96,930	37,146	49,993	1,410	n.d.	n.d.	n.d.	8,132	n.d.	0,249	n.d.	n.d.	n.d.	0,229	0,324	n.d.	n.d.	n.d.	n.d.	n.d.
**s.d.**		47,544	29,949	31,737	0,417				11,736		0,255				0,350	0,340					

Data level	epoxy-CBZ	Diazepam DAP	Alprazolam APL	Ranitidine RNT	Sotalol SOL	Dimetridazole DMZ	Acetaminophen AAP	Ibuprofen IPF	2OH-ibuprofen	Hydrochlorothiazide HCT	Ketoprofen KPF	Valsartan VLS	Naproxen NPX	Salicylic acid SAA
	Pharmaceuticals (PhACs)													
**Water**	0,389	0,120	0,134	0,467	3,026	1,378	72,858	89,649	521,562	12,887	42,266	2,342	32,246	11,833
**s.d.**	0,436	0,176	0,181	0,710	3,840	2,377	65,005	81,777	725,003	18,855	40,174	4,147	41,733	8,552
**Biofilm**	n.d.	n.d.	n.d.	n.d.	0,364	n.d.	n.d.	42,924	n.d.	0,211	51,201	n.d.	n.d.	12,830
**s.d.**					1,142			59,281		0,516	77,880			7,147
**Aquatic insects - total**	n.d.	n.d.	n.d.	n.d.	n.d.	n.d.	n.d.	n.d.	n.d.	n.d.	28,302	n.d.	5,675	29,917
**s.d.**											32,084		23,090	35,483
**Aquatic insects - aquatic**	n.d.	n.d.	n.d.	n.d.	n.d.	n.d.	n.d.	n.d.	n.d.	n.d.	18,628	n.d.	10,348	40,401
**s.d.**											26,229		30,523	42,330
**Aquatic insects - terrestrial**	n.d.	n.d.	n.d.	n.d.	n.d.	n.d.	n.d.	n.d.	n.d.	n.d.	40,049	n.d.	n.d.	17,187
**s.d.**											34,828			18,364
**Odonata total**	n.d.	n.d.	n.d.	n.d.	n.d.	n.d.	n.d.	n.d.	n.d.	n.d.	38,952	n.d.	7,537	19,294
**s.d.**											32,248		27,697	16,832
**Odonata aquatic**	n.d.	n.d.	n.d.	n.d.	n.d.	n.d.	n.d.	n.d.	n.d.	n.d.	28,489	n.d.	15,911	23,706
**s.d.**											24,110		38,909	11,915
**Odonata terrestrial**	n.d.	n.d.	n.d.	n.d.	n.d.	n.d.	n.d.	n.d.	n.d.	n.d.	49,216	n.d.	n.d.	17,025
**s.d.**											37,882			19,993
**Trichoptera total**	n.d.	n.d.	n.d.	n.d.	n.d.	n.d.	n.d.	n.d.	n.d.	n.d.	10,215	n.d.	2,805	46,193
**s.d.**											22,929		12,801	48,272
**Trichoptera aquatic**	n.d.	n.d.	n.d.	n.d.	n.d.	n.d.	n.d.	n.d.	n.d.	n.d.	1,490	n.d.	8,182	34,514
**s.d.**											5,161		21,413	18,948
**Trichoptera terrestrial**	n.d.	n.d.	n.d.	n.d.	n.d.	n.d.	n.d.	n.d.	n.d.	n.d.	19,251	n.d.	n.d.	23,833
**s.d.**											21,236			13,288
**Riparian spiders**	n.d.	n.d.	n.d.	n.d.	n.d.	n.d.	n.d.	n.d.	n.d.	n.d.	17,006	n.d.	0,732	13,792
**s.d.**											21,319		2,195	7,295

Data level	1H-benzotriazole 1HB	Tolyltriazole TOT	Caffeine CFN	Tris(2-butoxyethyl)phosphate TBEP	Tris (1-chloro-2-propyl)phosphate TCPP	Tris(2-carboxyethyl)phosphine TCEP	Bisphenol-A BPA	Estriol E3	Estrone E1	Progesterone PRG	Triclosan TCL	Methyparaben MPB	Ethyparaben EPB	Propylparaben PPB
	Endocrine disrupting compounds (EDCs)													
**Water**	16,534	3,131	181,673	24,075	19,764	4,025	3,541	n.d.	0,422	n.d.	0,031	2,437	0,654	0,450
**s.d.**	20,172	3,527	199,622	23,015	13,705	5,138	3,443		0,493		0,069	2,082	0,808	0,811
**Biofilm**	8,110	0,010	17,160	5,522	37,777	0,765	2,401	3,449	0,025	0,038	0,030	2,696	0,012	0,262
**s.d.**	10,188	0,022	7,757	7,002	46,311	1,171	2,124	6,786	0,056	0,087	0,080	2,345	0,035	0,219
**Aquatic insects - total**	56,425	n.d.	19,238	8,006	19,920	1,759	6,997	3,781	n.d.	0,246	0,176	5,526	0,737	0,853
**s.d.**	85,823		32,708	13,745	36,406	3,335	6,836	6,161		1,117	0,425	4,446	1,782	0,937
**Aquatic insects - aquatic**	77,340	n.d.	28,854	6,072	30,720	2,198	6,616	4,658	n.d.	n.d.	0,199	7,284	0,962	1,095
**s.d.**	109,963		40,914	12,315	46,377	4,315	6,581	7,443			0,546	4,883	2,294	1,161
**Aquatic insects - terrestrial**	31,028	n.d.	7,562	10,355	6,806	1,226	7,459	2,717	n.d.	0,545	0,148	3,390	0,469	0,560
**s.d.**	24,011		10,217	15,121	5,455	1,312	7,186	3,936		1,622	0,202	2,600	0,791	0,404
**Odonata total**	73,471	n.d.	25,669	10,953	28,715	2,520	8,259	1,975	n.d.	0,029	0,202	3,987	0,195	0,980
**s.d.**	103,998		39,728	14,557	44,110	4,033	8,018	5,638		0,125	0,316	3,856	0,304	1,136
**Odonata aquatic**	131,460	n.d.	51,647	10,940	54,326	4,151	8,452	3,463	n.d.	n.d.	0,200	5,967	0,199	1,609
**s.d.**	128,152		45,199	15,327	53,644	5,233	7,903	7,846			0,404	4,567	0,388	1,365
**Odonata terrestrial**	18,319	n.d.	2,542	9,341	4,904	1,168	8,983	0,706	n.d.	0,061	0,226	2,449	0,213	0,459
**s.d.**	12,079		4,921	13,934	3,589	1,503	8,218	1,573		0,178	0,215	1,673	0,211	0,330
**Trichoptera total**	30,277	n.d.	9,315	3,437	6,166	0,570	4,984	6,830	n.d.	0,607	0,152	8,030	1,516	0,669
**s.d.**	28,845		10,658	11,144	6,746	0,888	3,697	5,907		1,770	0,593	4,310	2,486	0,415
**Trichoptera aquatic**	10,017	n.d.	2,152	0,873	5,602	n.d.	3,259	6,269	n.d.	n.d.	n.d.	7,777	1,244	0,404
**s.d.**	11,999		3,981	3,023	6,872		3,764	7,471				4,055	2,497	0,295
**Trichoptera terrestrial**	55,398	n.d.	20,746	8,834	9,657	1,663	5,894	7,920	n.d.	1,770	0,010	6,355	1,163	0,926
**s.d.**	26,111		8,049	18,029	7,335	0,675	3,051	3,230		2,727	0,025	1,918	1,216	0,308
**Riparian spiders**	15,031	n.d.	5,013	9,327	4,382	n.d.	6,930	3,582	n.d.	n.d.	n.d.	3,712	0,423	0,527
**s.d.**	7,484		6,422	19,667	6,053		3,102	6,594				1,493	0,584	0,242

Table S6. Significance of differences of total concentrations and concentrations of individual compounds (pharmaceuticals [PhACs] and endocrine disrupting compounds [EDCs]) between aquatic insects and riparian spiders and larvae and adults of Odonata and Trichoptera according to the Mann-Whitney U tests. Significant p values are shown in red. Full names of compounds and abbreviations are listed in Table S5.

	Aquatic insects - riparian spiders		Odonata larvae - adults		Trichoptera larvae - adults	
**Totals**	**Mann-Whitney U**	**p-value**	**Mann-Whitney U**	**p-value**	**Mann-Whitney U**	**p-value**
**ECs**	124	0,0005	87	< 0.0001	53	0,2855
**PhACs**	137	0,0010	357	0,9036	46	0,1410
**EDCs**	251	0,0520	75	< 0.0001	0	< 0.0001
**Individual compounds**	**Mann-Whitney U**	**p-value**	**Mann-Whitney U**	**p-value**	**Mann-Whitney U**	**p-value**
**AZM**	340	0,3777	270	0,1038	44	0,1124
**SUM**	138	< 0.0001	n.a.	n.a.	n.a.	n.a.
**TIM**	333	0,3301	182	0,0015	62	0,5629
**GLC**	306	0,1848	310	0,3467	18	0,0012
**TIB**	273	0,0552	162	< 0.0001	14	0,0006
**CAZ**	360	0,2520	270	0,0053	42	0,0165
**KPF**	337,5	0,3525	234	0,0244	19	0,0010
**NPX**	410	0,9297	256,5	0,0026	60	0,1662
**SAA**	273	0,0926	211	0,0081	50	0,2144
**1HB**	273	0,0915	159	0,0004	0	< 0.0001
**CFN**	306	0,1775	27	< 0.0001	2	< 0.0001
**TBEP**	387	0,7402	332	0,5789	38	0,0193
**TCPP**	264	0,0721	100,5	< 0.0001	51	0,2241
**TCEP**	220,5	0,0104	213	0,0065	0	< 0.0001
**BPA**	354	0,4725	355	0,8756	45	0,1231
**PRG**	373,5	0,3286	324	0,0813	36	0,0071
**E3**	396	0,8067	347,5	0,6949	57	0,3989
**TCL**	229,5	0,0134	285	0,1637	60	0,1662
**MPB**	324	0,2824	200	0,0043	57	0,4025
**EPB**	363	0,5068	285	0,1198	24	0,0044
**PPB**	350	0,4438	187	0,0020	16	0,0013

Table S7. Significance of differences of total concentrations and concentrations of individual compounds (pharmaceuticals [PhACs] and endocrine disrupting compounds [EDCs]) between water (Wat) and biotic compartments in food webs at 3 sites in NW Croatia according to the Kruskal-Wallis ANOVA and post hoc tests. Biotic compartments; Bio-biofilm, AqLv - aquatic caddisfly larvae, TePy-terrestrial prey, adult caddisflies, TePd-terrestrial predators;spiders/dragonflies; significant p values are shown in red. Full names of compounds and abbreviations are listed in Table S5.

	Median Test				Kruskal-Wallis ANOVA by Ranks							
	Overall Median	Chi-Square	df	*p*	H (4, N= 60)	*p*	Multiple comparisons					
**Total ECs**	258,0620	2,6667	4	0,6151	7,7165	0,1025						
**Total PhACs**	90,3959	22,0000	4	0,0002	15,3931	0,0040		Wat	Bio	AqLv	TePy	TePd
							Wat		1,0000	1,0000	1,0000	0,0228
							Bio	1,0000		1,0000	1,0000	0,0854
							AqLv	1,0000	1,0000		1,0000	0,0033
							TePy	1,0000	1,0000	1,0000		0,4439
							TePd	0,0228	0,0854	0,0033	0,4439	
**Total EDCs**	68,6987	24,0000	4	0,0001	26,2426	<0.0001		Wat	Bio	AqLv	TePy	TePd
							Wat		1,0000	0,1605	0,2065	1,0000
							Bio	1,0000		0,0121	1,0000	0,4824
							AqLv	0,1605	0,0121		0,0000	1,0000
							TePy	0,2065	1,0000	0,0000		0,0054
							TePd	1,0000	0,4824	1,0000	0,0054	
	Median Test				Kruskal-Wallis ANOVA by Ranks							
**Individual compounds**	Overall Median	Chi-Square	df	*p*	H (4, N= 60)	*p*	Multiple comparisons					
**AZM**	1,2920	15,3333	4	0,0041	21,5357	0,0002		Wat	Bio	AqLv	TePy	TePd
							Wat		0,0029	0,0257	0,0003	0,0094
							Bio	0,0029		1,0000	1,0000	1,0000
							AqLv	0,0257	1,0000		1,0000	1,0000
							TePy	0,0003	1,0000	1,0000		1,0000
							TePd	0,0094	1,0000	1,0000	1,0000	
**SUM**	0,0000	26,6667	4	<0.0001	26,0000	<0.0001		Wat	Bio	AqLv	TePy	TePd
							Wat		1,0000	1,0000	1,0000	0,3539
							Bio	1,0000		1,0000	1,0000	0,3539
							AqLv	1,0000	1,0000		1,0000	0,3539
							TePy	1,0000	1,0000	1,0000		0,3539
							TePd	0,3539	0,3539	0,3539	0,3539	
**TIM**	0,3526	18,6667	4	0,0009	24,5345	0,0001		Wat	Bio	AqLv	TePy	TePd
							Wat		0,0000	0,0288	0,0247	0,3340
							Bio	0,0000		0,7660	0,8471	0,0869
							AqLv	0,0288	0,7660		1,0000	1,0000
							TePy	0,0247	0,8471	1,0000		1,0000
							TePd	0,3340	0,0869	1,0000	1,0000	
**GLC**	0,0000	20,5263	4	0,0004	21,9843	0,0002		Wat	Bio	AqLv	TePy	TePd
							Wat		1,0000	1,0000	0,0027	0,2336
							Bio	1,0000		1,0000	0,0931	1,0000
							AqLv	1,0000	1,0000		0,0382	1,0000
							TePy	0,0027	0,0931	0,0382		1,0000
							TePd	0,2336	1,0000	1,0000	1,0000	
**TIB**	0,0000	35,2232	4	<0.0001	37,8176	<0.0001		Wat	Bio	AqLv	TePy	TePd
							Wat		1,0000	1,0000	0,0005	0,0476
							Bio	1,0000		1,0000	0,0000	0,0059
							AqLv	1,0000	1,0000		0,0031	0,1851
							TePy	0,0005	0,0000	0,0031		1,0000
							TePd	0,0476	0,0059	0,1851	1,0000	
**CAZ**	0,0000	40,3977	4	<0.0001	43,2127	<0.0001		Wat	Bio	AqLv	TePy	TePd
							Wat		0,0003	0,0003	0,0693	0,0003
							Bio	0,0003		1,0000	1,0000	1,0000
							AqLv	0,0003	1,0000		1,0000	1,0000
							TePy	0,0693	1,0000	1,0000		1,0000
							TePd	0,0003	1,0000	1,0000	1,0000	
**KPF**	9,2235	11,3333	4	0,0231	4,1467	0,0068		Wat	Bio	AqLv	TePy	TePd
							Wat		1,0000	0,0368	1,0000	1,0000
							Bio	1,0000		0,3912	1,0000	1,0000
							AqLv	0,0368	0,3912		0,0305	0,0247
							TePy	1,0000	1,0000	0,0305		1,0000
							TePd	1,0000	1,0000	0,0247	1,0000	
**NPX**	0,0000	6,2963	4	0,1781	6,0164	0,1970						
**SAA**	17,0362	17,3333	4	0,0017	17,5992	0,0015		Wat	Bio	AqLv	TePy	TePd
							Wat		0,8577	0,0023	0,3151	1,0000
							Bio	0,8577		0,4958	1,0000	1,0000
							AqLv	0,0023	0,4958		1,0000	0,0083

								Wat	Bio	AqLv	TePy	TePd
							TePy	0,3151	1,0000	1,0000		0,7004
							TePd	1,0000	1,0000	0,0083	0,7004	
	11,0809	24,6667	4	0,0001	37,3495	<0.0001		Wat	Bio	AqLv	TePy	TePd
							Wat		1,0000	1,0000	0,0000	0,3590
**1HB**							Bio	1,0000		1,0000	0,0000	0,0706
							AqLv	1,0000	1,0000		0,0002	0,9697
							TePy	0,0000	0,0000	0,0002		0,0947
							TePd	0,3590	0,0706	0,9697	0,0947	
	11,2273	29,3333	4	<0.0001	33,8755	<0.0001		Wat	Bio	AqLv	TePy	TePd
							Wat		1,0000	0,0001	1,0000	0,0092
**CFN**							Bio	1,0000		0,0006	1,0000	0,0305
							AqLv	0,0001	0,0006		0,0003	1,0000
							TePy	1,0000	1,0000	0,0003		0,0184
							TePd	0,0092	0,0305	1,0000	0,0184	
	3,3970	12,0000	4	0,0174	15,2227	0,0043		Wat	Bio	AqLv	TePy	TePd
							Wat		1,0000	0,0121	1,0000	1,0000
**TBEP**							Bio	1,0000		0,0136	1,0000	1,0000
							AqLv	0,0121	0,0136		0,6479	0,1258
							TePy	1,0000	1,0000	0,6479		1,0000
							TePd	1,0000	1,0000	0,1258	1,0000	
	7,7042	8,6667	4	0,0700	12,8311	0,0121		Wat	Bio	AqLv	TePy	TePd
							Wat		1,0000	0,9464	1,0000	1,0000
**TCPP**							Bio	1,0000		0,0207	0,5676	0,0341
							AqLv	0,9464	0,0207		1,0000	1,0000
							TePy	1,0000	0,5676	1,0000		1,0000
							TePd	1,0000	0,0341	1,0000	1,0000	
	0,0000	40,5804	4	<0.0001	42,2792	<0.0001		Wat	Bio	AqLv	TePy	TePd
							Wat		0,0024	0,0000	1,0000	0,0003
**TCEP**							Bio	0,0024		1,0000	0,0293	1,0000
							AqLv	0,0000	1,0000		0,0010	1,0000
							TePy	1,0000	0,0293	0,0010		0,0052
							TePd	0,0003	1,0000	1,0000	0,0052	
	3,7581	16,6667	4	0,0022	19,5149	0,0006		Wat	Bio	AqLv	TePy	TePd
							Wat		1,0000	1,0000	0,1279	0,0102
**BPA**							Bio	1,0000		1,0000	0,0915	0,0067
							AqLv	1,0000	1,0000		0,4439	0,0503
							TePy	0,1279	0,0915	0,4439		1,0000
							TePd	0,0102	0,0067	0,0503	1,0000	
	0,0000	26,6667	4	<0.0001	26,1281	<0.0001		Wat	Bio	AqLv	TePy	TePd
							Wat		1,0000	1,0000	0,3539	1,0000
**PRG**							Bio	1,0000		1,0000	0,3539	1,0000
							AqLv	1,0000	1,0000		0,3539	1,0000
							TePy	0,3539	0,3539	0,3539		0,3539
							TePd	1,0000	1,0000	1,0000	0,3539	
	0,0000	28,7500	4	<0.0001	19,8968	0,0005		Wat	Bio	AqLv	TePy	TePd
							Wat		1,0000	0,2130	0,0021	1,0000
**E3**							Bio	1,0000		1,0000	0,1794	1,0000
							AqLv	0,2130	1,0000		1,0000	1,0000
							TePy	0,0021	0,1794	1,0000		0,0840
							TePd	1,0000	1,0000	1,0000	0,0840	
	0,0000	14,8296	4	0,0051	10,8282	0,0286		Wat	Bio	AqLv	TePy	TePd
							Wat		1,0000	0,1238	0,8577	1,0000
**TCL**							Bio	1,0000		1,0000	1,0000	1,0000
							AqLv	0,1238	1,0000		1,0000	1,0000
							TePy	0,8577	1,0000	1,0000		1,0000
							TePd	1,0000	1,0000	1,0000	1,0000	
	3,9083	15,3333	4	0,0041	25,3027	<0.0001		Wat	Bio	AqLv	TePy	TePd
							Wat		0,2717	0,0001	0,0008	1,0000
**MPB**							Bio	0,2717		0,3244	0,8159	1,0000
							AqLv	0,0001	0,3244		1,0000	0,0581
							TePy	0,0008	0,8159	1,0000		0,1823
							TePd	1,0000	1,0000	0,0581	0,1823	
	0,0535	32,6667	4	<0.0001	27,8481	<0.0001		Wat	Bio	AqLv	TePy	TePd
							Wat		0,0947	1,0000	0,4317	1,0000
**EPB**							Bio	0,0947		1,0000	0,0000	0,0884
							AqLv	1,0000	1,0000		0,0035	1,0000
							TePy	0,4317	0,0000	0,0035		0,4564
							TePd	1,0000	0,0884	1,0000	0,4564	
	0,4267	17,3333	4	0,0017	22,6691	0,0001		Wat	Bio	AqLv	TePy	TePd

PPB

Wat		1,0000	1,0000	0,0018	0,9349
Bio	1,0000		1,0000	0,0002	0,2637
AqLv	1,0000	1,0000		0,0411	1,0000
TePy	0,0018	0,0002	0,0411		0,3857
TePd	0,9349	0,2637	1,0000	0,3857	
